# Akt1 and dCIZ1 promote cell survival from apoptotic caspase activation during regeneration and oncogenic overgrowth

**DOI:** 10.1038/s41467-020-19068-2

**Published:** 2020-11-12

**Authors:** Gongping Sun, Xun Ding, Yewubdar Argaw, Xiaoran Guo, Denise J. Montell

**Affiliations:** 1 The Key Laboratory of Experimental Teratology, Ministry of Education and Department of Anatomy and Histoembryology, School of Basic Medical Sciences, Cheeloo College of Medicine, Shandong University, Jinan, Shandong China; 2 Molecular, Cellular, and Developmental Biology Department, University of California, Santa Barbara, CA 93106 USA

**Keywords:** Oncogenes, Apoptosis, Stress signalling

## Abstract

Apoptosis is an ancient and evolutionarily conserved cell suicide program. During apoptosis, executioner caspase enzyme activation has been considered a point of no return. However, emerging evidence suggests that some cells can survive caspase activation following exposure to apoptosis-inducing stresses, raising questions as to the physiological significance and underlying molecular mechanisms of this unexpected phenomenon. Here, we show that, following severe tissue injury, Drosophila wing disc cells that survive executioner caspase activation contribute to tissue regeneration. Through RNAi screening, we identify *akt1* and a previously uncharacterized Drosophila gene *CG8108*, which is homologous to the human gene CIZ1, as essential for survival from the executioner caspase activation. We also show that cells expressing activated oncogenes experience apoptotic caspase activation, and that Akt1 and dCIZ1 are required for their survival and overgrowth. Thus, survival following executioner caspase activation is a normal tissue repair mechanism usurped to promote oncogene-driven overgrowth.

## Abstract (web summary)

Although executioner caspase activation is considered terminal, some cells are capable of survival, suggesting additional regulation. Here, the authors show that cells in the Drosophila wing imaginal disc survive caspase activation via Akt1 and dCIZ1 and actively participate in tissue regeneration.

## Introduction

Precise control of cell death and survival is critical for tissue homeostasis. Apoptosis is an important form of programmed cell death executed by proteolytic enzymes called caspases. While physiological apoptosis promotes normal development and homeostasis, inappropriate apoptosis causes tissue damage, degenerative diseases, and immune system dysfunction^1^.

**Fig. 1:**
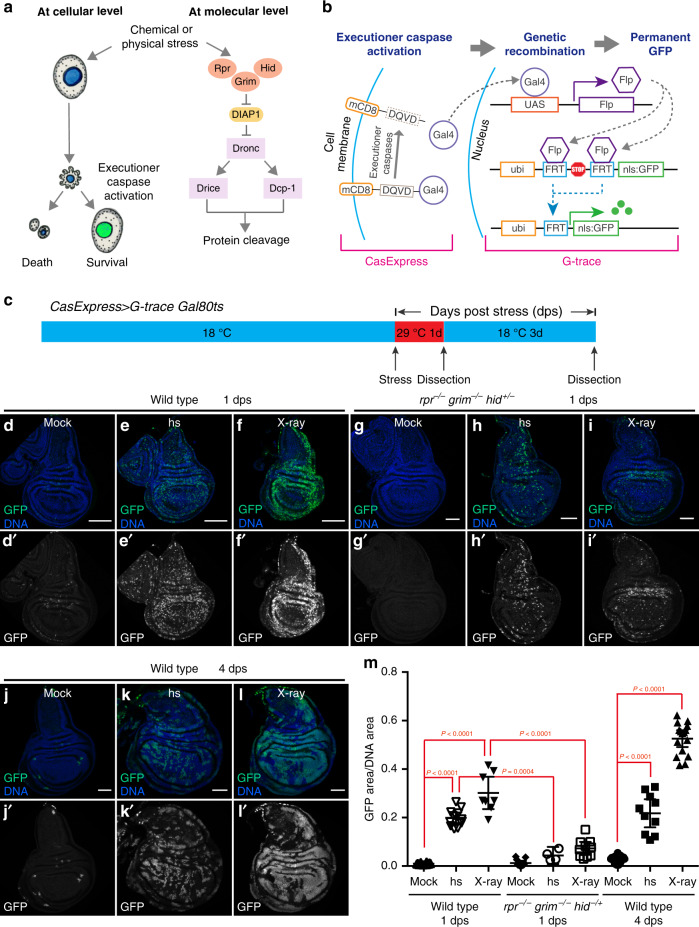
Survival from executioner caspase activation contributes to regeneration after heat shock or X-ray irradiation. (**a**) A schematic representation of cellular and molecular events in cells that experience stress-induced executioner caspase activation. (**b**) A schematic of CasExpress and G-trace. (**c**) A schematic of the experimental design for results shown in (**d**–**m**). (**d**–**f**) CasExpress activation (GFP) in wild type wing discs after mock treatment (**d**), heat shock (hs) (**e**), or radiation (X-ray) (**f**) and 1d at 29 °C. (**g**–**i**) CasExpress activation (GFP) in *rpr*^*−/−*^ *grim*^*−/−*^ *hid*^*−/+*^ wing discs after mock treatment (**g**), heat shock (**h**), or radiation (**i**) and 1d at 29 °C. (**j**–**l**) CasExpress activation (GFP) in wild type wing discs after mock treatment (**j**), heat shock (**k**), or radiation (**l**), 1d at 29 °C, and 3d at 18 °C. All scale bars represent 50 μm. (**m**) Quantification of the percentage of GFP^+^ cells in discs in (**d**-**l**). *n* = 12 (wild type 1dps mock), 12 (wild type 1dps hs), 8 (wild type 1dps X-ray), 11 (*rpr*^*−/−*^*grim*^*−/−*^*hid*^*−/+*^ 1dps mock), 4 (*rpr*^*−/−*^*grim*^*−/−*^*hid*^*−/+*^ 1dps hs), 10 (*rpr*^*−/−*^*grim*^*−/−*^*hid*^*−/+*^ 1dps X-ray), 13 (wild type 4dps mock), 10 (wild type 4dps hs), 16 (wild type 4dps X-ray). *n* is the number of biological independent samples used for quantification. The data are presented as mean values ± 95% confidence interval. Statistical significance was determined after the logarithm transformation using one-way ANOVA. The Tukey test was used to derive adjusted *P*-value for multiple comparisons. Source data are provided as a Source Data file.

In the process of apoptosis, activation of executioner caspases results in cleavage of hundreds of proteins, thereby dismantling the cell. Executioner caspase activation has thus been considered a point of no return^2^. However, recent studies show that some cells can survive apoptosis-inducing stresses even after executioner caspase activation^3–8^. Cultured mammalian cells can recover from executioner caspase activation induced by transient exposure to apoptotic inducers like ethanol, staurosporine, and TNFα, through a process called anastasis^3,4,6^. Similar events have been described in tumors cells after tBid overexpression^5^ or treatment with chemotherapeutic drugs like paclitaxel^8^. Survival after stress-induced executioner caspase activation has also been reported in cardiomyocytes after ischemia-reperfusion^9^ and in neurons expressing pathogenic tau^10^ in mice. These studies suggest that, in addition to the established competition between pro-death and pro-survival signals upstream of caspases, there is another layer of regulation after executioner caspases activation (Fig. 1a). Deciphering these regulatory mechanisms could therefore identify new therapeutic targets for a variety of diseases caused by improper cell death versus survival decisions.

To identify cells that survive executioner caspase activation in vivo, we previously developed a biosensor, CasExpress, which labels cells that have experienced transient caspase activation in Drosophila (Fig. 1b)^11^. We discovered widespread survival after executioner caspase activation during normal development of Drosophila larval and adult tissues including rapidly proliferating larval imaginal discs^11^. A similar sensor exhibits similar activation in adult tissues^12^. Here, we use the CasExpress sensor to decipher the physiological importance of, and molecular mechanisms enabling, survival from stress-induced executioner caspase activation in vivo. We show that although executioner caspase activation induced by heat shock, X-ray irradiation, or transient pro-apoptotic gene overexpression causes extensive cell death in wing imaginal discs, many epithelial cells survive and participate in tissue regeneration. We also report that wing disc epithelial cells experience transient apoptosis-inducing stresses even during normal development. To investigate the molecular mechanisms enabling cell survival from transient caspase activation, we developed a second-generation CasExpress sensor compatible with RNAi screening. We identified Akt1 and a new gene, CG8108 (dCIZ1), as essential for survival from executioner caspase activation. Finally, we show that cells expressing activated oncogenes experience homeostatic apoptotic stress and exploit Akt1 and dCIZ1 to survive. Thus, survival from executioner caspase activation is a normal cellular response to tissue injury that promotes repair and can be hijacked to drive tumor growth.

## Results

### Survival from heat shock- or X-irradiation-induced executioner caspase activation contributes to tissue regeneration

The observations that a variety of cells can survive executioner caspase activation in response to diverse stresses (Fig. 1a) raises the question as to the physiological significance of this unexpected cellular resilience. We hypothesized that it might represent an injury repair mechanism. To test whether cells can survive executioner caspase activation after tissue damage in vivo, we exposed Drosophila larvae to heat shock and radiation, which are known to cause massive apoptosis in wing imaginal discs^13^. To assess whether any cells survived executioner caspase activation, we took advantage of the CasExpress biosensor^11^. CasExpress is a fusion protein composed of the extracellular and transmembrane domains of mCD8, 200 amino acids from an endogenous executioner caspase target protein containing the proteolytic cleavage site (DQVD), and the transcription factor Gal4 (Fig. 1b). Upon caspase cleavage, Gal4 is released and translocates to the nucleus where, in combination with the G-trace lineage tracing system^14^, it catalyzes DNA recombination that results in permanent GFP expression in that cell and all of its progeny^11^. In the presence of Gal80^ts^, Gal4 activity is repressed at 18 °C. We grew animals at 18 °C to the mid-third instar larval stage to silence reporter activity during normal development. Larvae were then exposed to 2 hrs heat shock at 37 °C or 10 Gy X-ray and transferred to 29 °C to allow Gal4 to function (Fig. 1c). After 24 h at 29 °C, we stained for cleaved Dcp-1 to detect dead cells. We note that cleaved Dcp-1 (cDcp1), which is an active form of the executioner caspase Dcp-1, accumulates detectably only in dead cells. In living/recovering cells, active caspase turns over rapidly and does not accumulate^15^. Moreover, dead/dying cells shrink, further concentrating the signal in small, intense clusters. Dead cells are also extruded and thus are primarily located in a different focal plane from the living epithelium. We confirmed that extensive apoptotic cell death occurs in the discs of irradiated or heat-shocked larvae^13^ (Supplementary Figure S1a–c).

To determine whether any cells that activated executioner caspase survived, we examined CasExpress flies for GFP expression. One day after stress, ~20% of cells in discs from heat-shocked larvae and ~30% of cells in the discs from irradiated animals were GFP^+^ (CasExpress^+^), compared to 0.9% in the mock-treated controls (Fig. 1d–f, m). Discs expressing a caspase-insensitive control sensor, in which the executioner caspase cleavage site DQVD is mutated to DQVA, showed no GFP^+^ cells after stress (Supplementary Figure S1d–f). The stress-induced CasExpress activation was blocked in discs that lacked the initiator caspase Dronc (Supplementary Figure S1g–i). We then tested whether caspase activation in the GFP^+^ cells required the pro-apoptotic proteins Reaper (Rpr), Head involution defective (Hid), and Grim (Fig. 1a). Drosophila lacking *rpr*, *hid*, and *grim* die as embryos^16^, precluding analysis of triple mutant larvae. Animals homozygous for *rpr* and *grim* and heterozygous for *hid* are viable to larval stages and exhibited a significant reduction in the percentage of GFP^+^ cells after stress (Fig. 1g–i, m), indicating that CasExpress activation depends on these initiators of apoptosis.

To determine whether the GFP^+^ cells in the stressed discs ultimately contributed to the regenerated discs, after stress and one day at 29 °C, we transferred the larvae back to 18 °C for an additional 3 days recovery (Fig. 1c). At the time of dissection, all GFP^+^ cells should be the progeny of cells that activated executioner caspase during the one day at 29 °C. Four days after stress, the discs exhibited normal morphology (Supplementary Figures S1j–l), and the cDcp1^+^ dead cells in the discs were diminished (compare Supplementary Figures S1m–o to Supplementary Figures S1a–c), indicating the discs had regenerated. Importantly, a large proportion of the regenerated discs were GFP^+^ (Fig. 1j–m), and some of the GFP^+^ cells were proliferating, demonstrated by co-localization of GFP and phospho-histone H3 (PH3) staining (Supplementary Figure S1p). Therefore, we conclude that cells that survived stress-induced executioner caspase activation contributed to tissue regeneration following injury.

To determine whether cells that survived stress-induced executioner caspase activation were capable of differentiating, we examined regenerated eye discs after radiation. In early larval stages, like wing discs, eye disc cells are proliferative. At the beginning of the third instar, cells begin to differentiate into multiple cell types including photoreceptor neurons. We irradiated larvae carrying *CasExpress*, *G-trace,* and *Gal80*^*ts*^, and transferred them to 29 °C for 1day to allow Gal4 to function prior to the initiation of differentiation, and then returned them to 18 °C (Supplementary Figure S1q). After 4 days at 18 °C, we found a large proportion of differentiated neurons (elav^+^) were GFP^+^ cells (Supplementary Figure S1s), indicating that cells that survived X-ray-induced executioner caspase activation can differentiate normally.

### Cells can survive *rpr*-induced executioner caspase activation

**Fig. 2:**
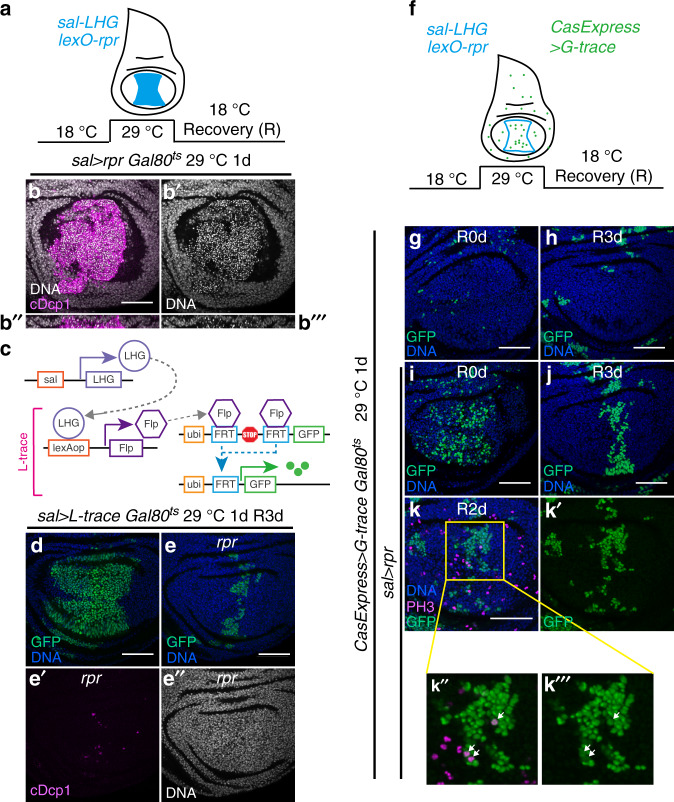
Cells can survive *rpr*-induced executioner caspase activation. (**a**) A schematic showing the *spalt* (*sal*) domain (blue region) and the design for experiments in (**b**, **d**, and **e**). Transgenes were expressed only at 29 °C. (**b**) Overexpression of *rpr* for 1d induced extensive apoptotic cell death (cDcp1 staining and pyknotic nuclei). (**b”**) and (**b”’**) show the vertical sections through the disc in (**b**). (**c**) A schematic shows the use of L-trace to trace *LHG*-expressing cells. (**d**) *sal-LHG L-trace Gal80*^*ts*^ disc after 1d at 29 °C and 3d at 18 °C. GFP labels cells descended from the *sal-LHG* domain. (**e**) *sal-LHG lexO-rpr L-trace Gal80*^*ts*^ disc after 1d at 29 °C and 3d at 18 °C. GFP labels cells have experienced transient *rpr* overexpression and their progeny. (**f**) A schematic of experiments in (**g**–**k**). The blue line circles the *sal* domain. (**g**, **h**) *lexO-rpr CasExpress G-trace Gal80*^*ts*^ disc right after 1d at 29 °C (**g**) and after 3d recovery at 18 °C (**h**). (**i**–**k**) *sal-LHG lexO-rpr CasExpress G-trace Gal80*^*ts*^ disc right after 1d at 29 °C (**i**), after 3d recovery at 18 °C (**j**), and after 2d recovery at 18 °C (**k**). In (**g**–**k**), GFP marks cells that have survive executioner caspase activation. In (**k**), PH3 labels mitotic cells. White arrows point to several examples of mitotic CasExpress^+^ cells. In all images, scale bar is 50 μm.

To test whether cells can recover from more direct activation of the apoptotic pathway caused by overexpression of the pro-apoptotic protein Rpr, we used the LexA/lexO transcription system to drive *rpr* specifically in the central *spalt* (*sal*) domain of the wing pouch (Fig. 2a). We used a Gal80-regulatable version of LexA, LHG, and Gal80^ts^, to control the timing of *rpr* overexpression^17,18^ (Fig. 2a). As expected, discs overexpressing *rpr* for one day exhibited intense executioner caspase activation and cell death, as shown by accumulation of cells with cDcp1 and pyknotic nuclei (Fig. 2b–b”’). To test if any *rpr*-overexpressing cells survived, we used L-trace (Fig. 2c) which, similar to G-trace, permanently labels cells that originate from the *sal-LHG*-expressing region with GFP. After *rpr* overexpression for one day at 29 °C, larvae were transferred back to 18 °C for recovery. Three days later, the dead cells had been mostly eliminated and normal disc morphology restored (Fig. 2e-e”). A large fraction of the *rpr*-overexpressing cells died and were eliminated from the epithelium (compare Fig. 2e to d). However, the presence of GFP^+^ cells in the restored epithelium (Fig. 2e) indicated that a fraction of cells can survive transient *rpr* overexpression.

To assess whether these survivors had experienced executioner caspase activation or had simply escaped either *rpr* expression or caspase activation, we used CasExpress and G-trace to monitor survival from executioner caspase activation following *rpr* overexpression (Fig. 2f). Compared to controls in which *rpr* was not overexpressed (Fig. 2g), we found increased CasExpress^+^ (GFP^+^) cells in the recovered epithelium after one day of *rpr* overexpression at 29 °C (Fig. 2i). After 3 days regeneration at 18 °C, few CasExpress^+^ cells were present in the control without *rpr* overexpression (Fig. 2h), compared to the regenerated epithelium (Fig. 2j). The CasExpress^+^ cells in the regenerated epithelium were at the same location (middle of the pouch) and abundance as those that survived transient *rpr* overexpression (compare Fig. 2j to e), indicating that most, if not all, *rpr*-overexpressing cells experienced executioner caspase activation. While many cells died, some survived and remained in the regenerated discs. After 2 days of regeneration, phospho-histone H3 (PH3) staining showed actively proliferating CasExpress^+^ cells (Fig. 2k-k”’).

### Developmental CasExpress activation depends on apoptosis-inducing signals

**Fig. 3:**
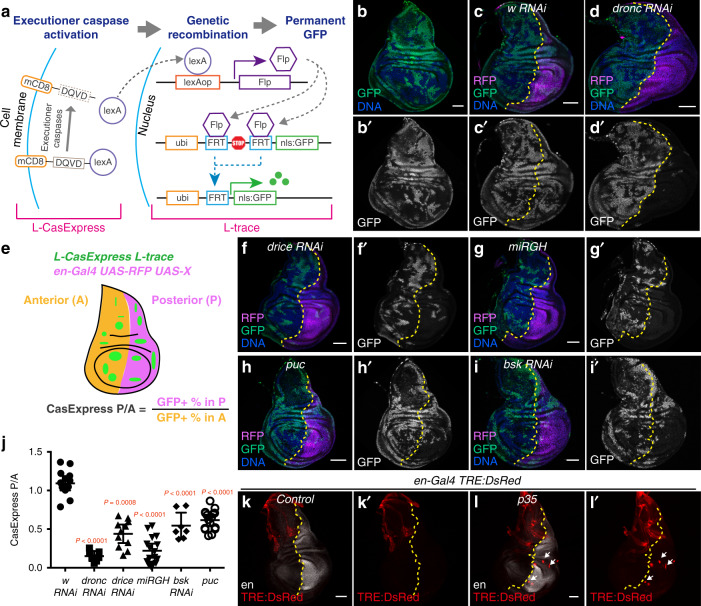
Developmental CasExpress activation depends on apoptosis-inducing signals. (**a**) A schematic of L-CasExpress and L-trace. (**b**) *L-CasExpress; L-trace* wing disc. GFP labels cells that survive executioner caspase activation. (**c**, **d**) Discs expressing *enGal4*, *UAS-RFP*, *L-CasExpress*, *L-trace*, and *UAS-w RNAi* (**c**) or *UAS-dronc RNAi* (**d**). (**e**) A schematic of the method for quantifying the effect of a genetic manipulation on survival from executioner caspase activation. A transgene (X) is expressed in the posterior compartment of the wing disc under *en-Gal4*, and survival from executioner caspase activation in the whole disc is monitored by L-CasExpress. The effect of expression of X on CasExpress activation is measured by the ratio of the percentage of GFP^+^ cells in the posterior compartment to that in the anterior compartment. (**f**–**i**) The effect of inhibiting apoptosis or JNK pathway on cell survival. Wing discs shown in the images expressed *L-CasExpress*, *L-trace*, *en-Gal4*, *UAS-RFP*, and *UAS-drice RNAi* (**f**), *UAS-miRGH* (**g**), *UAS-puc* (**h**), or *UAS-bsk RNAi* (**i**). (**j**) Quantification of CasExpress P/A ratio of the listed genotypes. *n* = 14 (*w RNAi*), 7 (*dronc RNAi*), 11 (*drice RNAi*), 16 (*miRGH*), 7 (*bsk RNAi*), 16 (*puc*). n is the number of biological independent samples used for quantification. The data are presented as mean values ± 95% confidence interval. Statistical significance was determined after the logarithm transformation using one-way ANOVA. The Tukey test was used to derive adjusted *P*-value for multiple comparisons. The P values compared to *w RNAi* are shown in the plot. Source data are provided as a Source Data file. (**k**, **l**) More cells with JNK activity were kept in the epithelium when execution of apoptosis was blocked (white arrows in **l**). Trachea attached to the disc are also DsRed^+^ and not affected by p35 expression. In all images, RFP marks *UAS* transgene expressing region. The yellow dotted lines mark the boundary between the anterior compartment and the posterior compartment. Scale bar is 50 μm.

We previously showed that ~1% of cells in a growing disc activate CasExpress during any given 24-h period of normal disc development (Fig. 1d, m)^11^. Activation of CasExpress during normal development is dependent on the upstream caspase Dronc (Supplementary Figure S1g)^11^, and is thus distinct from the more recently described phenomenon of “basal” caspase activation in discs, which is Dronc-independent^19^. To gain further insight into the cellular and molecular mechanisms driving CasExpress activation, we set out to knockdown or overexpress genes and assess their effects on CasExpress. However, the original CasExpress employs Gal4/UAS and FLP/FRT, precluding Gal4/UAS-mediated RNAi or overexpression. To circumvent this limitation, we redesigned the CasExpress sensor. We replaced Gal4 with LexA and G-trace with L-trace, which is the LexA-responsive version of G-trace (Fig. 3a). Similar to the Gal4/UAS version, the LexA construct (henceforth referred to as L-CasExpress) showed a large fraction of GFP^+^ (CasExpress^+^) cells in third larval instar wing discs (Fig. 3b, b’). Although ~50% of disc cells are GFP^+^ by this stage, it is important to note that this reflects the sum of all cells that activate the sensor at any point in development and all of their progeny. Within any given 24 h period, only ~0.9% cells activate CasExpress (Fig. 1d, m).

To test L-CasExpress activation for Dronc dependence and establish a quantitative RNAi screening assay, we used *engrailed-Gal4* (*en-Gal4*) to express *UAS-dronc RNAi* specifically in the posterior compartment of the wing disc. The anterior compartment thus serves as an internal control. Compared to the *white* (*w*) *RNAi* control (Fig. 3c, c’), knocking down *dronc* blocked L-CasExpress activation (Fig. 3d, d’) indicating that L-CasExpress, like the original CasExpress, is Dronc-dependent. To quantify the effect of *dronc RNAi* (or any UAS transgene) on L-CasExpress activation, we calculated the ratio of the percentage of cells that were GFP^+^ in the posterior compartment to that in the anterior compartment (CasExpress P/A) (Fig. 3e, j).

Using this assay, we found overexpression of P35, a baculovirus protein that specifically binds and inhibits the executioner caspases, virtually abolished L-CasExpress activation (Supplementary Figure S2a, b). We further tested which of the two Drosophila executioner caspases, Drice and Dcp-1, is required for CasExpress activation. We found that *drice RNAi* reduced CasExpress activation (Fig. 3f, f’ and j), whereas loss of *dcp-1* had no measurable effect (Supplementary Figure S2c–e). This is notable because apoptosis induction in wing discs requires Drice but not Dcp-1^20,21^, whereas basal caspase activation depends on both^19^. Thus, CasExpress activation, like apoptotic induction^20^, reflects Drice activation during wing disc development.

Expression of *miRGH*, a microRNA that targets three pro-apoptotic genes *rpr*, *hid,* and *grim*^22,23^ also reduced CasExpress activation (Fig. 3g, g’, j). Animals homozygous for *rpr* and *grim*, and heterozygous for *hid*, exhibited a nearly 20-fold reduction in GFP^+^ imaginal disc cells (from 54 to 3%, Supplementary Figure S2f–h). Therefore, developmental CasExpress activation, like apoptosis—but unlike basal caspase activity^19^—depends on Rpr, Hid, and Grim, Dronc, and Drice, but not Dcp-1. Although some cell types, such as sperm, require the upstream apoptotic cascade and caspase activation for normal differentiation^24,25^, the patterns of L-CasExpress and such non-apoptotic caspase activation are different: Caspase is activated in every sperm cell at precisely the same stage of development^25^; whereas activation of CasExpress in the wing disc occurs in a subset of cells and is sporadic over time (ref. ^11^ and this study). Together, the data, therefore, suggest that CasExpress^+^ cells are cells that survive apoptotic executioner caspase activation during normal development.

These findings suggest that some cells sporadically experience endogenous, apoptosis-inducing stress during normal wing disc development, which is consistent with the fact that apoptotic cell death occurs in wing discs without any externally imposed stress^26^. To test this hypothesis, we inhibited Jun N-terminal kinase (JNK), a well-established stress-activated kinase^27^. Overexpression of the JNK inhibitor *puckered* (*puc*) (Fig. 3h, h’) or knockdown of the Drosophila JNK, called *basket* (*bsk*) (Fig. 3i, i’) reduced L-CasExpress activation (Fig. 3j). Using a JNK activity reporter, TRE:DsRed^28^, we found that active JNK was present in the scutellum region but barely detectable in the pouch or hinge regions of normal wing discs (Fig. 3k, k’). Overexpression of *p35*, which blocks the execution of apoptosis and thereby leads to the abnormal accumulation of stressed cells, caused an increase in the number of cells with activated JNK (Fig. 3l, l’), suggesting activated JNK normally eliminates stressed cells by apoptosis. These data together suggest that during development, transient apoptotic stresses activate JNK signaling, which in turn initiates executioner caspase activation. Cells that die are rapidly removed from the epithelium, while others survive as L-CasExpress^+^ cells.

### Identification of genes that regulate survival from stress-induced caspase activation

**Fig. 4:**
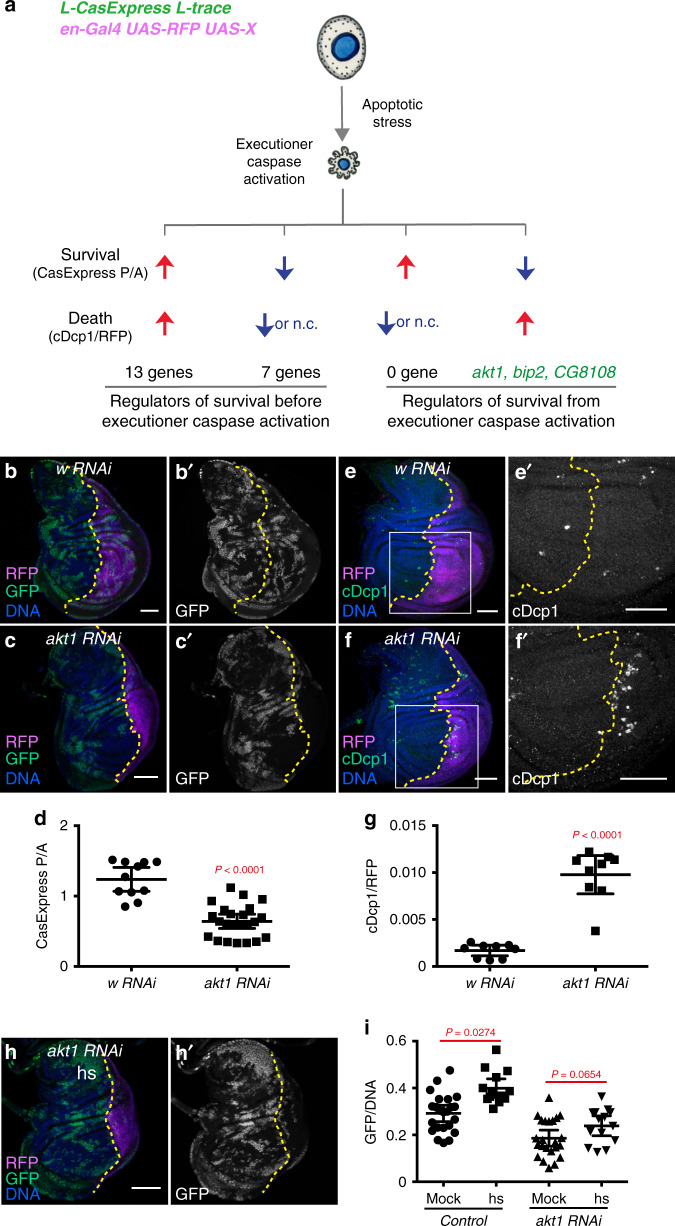
Identification of Akt1 as a promoter for survival from execution caspase activation through RNAi screening. (**a**) Classification and interpretations of RNAi effects on caspase activation followed by death (quantified by cDcp1^+^ area over RFP^+^ area, cDcp1/RFP) or survival (quantified by CasExpress percentage in posterior compartment over that in anterior compartment, CasExpress P/A). n.c.: no change. (**b**–**d**) Knocking down *akt1* for 3d suppressed CasExpress activation (GFP). The discs in (**b**) and (**c**) expressed *enGal4*, *UAS-RFP*, *L-CasExpress*, *L-trace*, *Gal80*^*ts*^ and *UAS-w RNAi* (**b**) or *UAS-akt1 RNAi* (**c**). For the quantification in (**d**), *n* = 11 (*w RNAi*), 23 (*akt1 RNAi*). (**e**–**g**) Knocking down *akt1* for 3d increased cell death (cDcp1^+^). The discs in (**e**) and (**f**) expressed *enGal4*, *UAS-RFP*, *Gal80*^*ts*^, and *UAS-w RNAi* (**e**) or *UAS-akt1 RNAi* (**f**). (**e’**) and (**f’**) are the magnified images of the white rectangular region in (**e**) and (**f**). For the quantification in (**g**), *n* = 9 for both genotypes. (**h**) Heat shock cannot induce CasExpress activation in *akt1*-knocked-down compartment. (**i**) Quantification of the percentage of GFP^+^ cells in the anterior compartment (control) and in the *akt1 RNAi* compartment in mock-treated or heat-shocked discs. *n* = 23 (mock), 14 (hs). In all images, RFP marks *UAS* transgene expressing region. The yellow dotted lines mark the boundary between the anterior compartment and the posterior compartment. Scale bar is 50 μm. In all dot plots, n is the number of biological independent samples used for quantification. The data are presented as mean values ± 95% confidence interval. ns: no statistical significance. Statistical significance was determined after logarithm transformation using unpaired two-tailed *t*-test. Source data are provided as a Source Data file.

A key unanswered question is what molecular mechanisms rescue cells from stress-induced executioner caspase activation. To address this question, we crossed *en-Gal4* and *UAS-RNAi* lines, together with the newly developed *L-CasExpress* sensor and L-trace, to screen for genes that enhanced or suppressed survival from stress-induced executioner caspase activation during development (Fig. 4a). We reasoned that genes associated with stress responses, growth, survival, and regeneration^29^ would be good candidates.

We measured the percentage of cells with CasExpress activation in the *RNAi*-expressing compartment compared to the percentage in the control compartment (CasExpress P/A) (Fig. 3e). By taking the percentage, we normalized for general effects on growth and survival since these should equally affect GFP^+^ and GFP^−^ cells. The percentage of GFP^+^ cells measures the frequency of survival from executioner caspase activation. A change in CasExpress activation could in principle result either from a change in the fraction of cells that activate executioner caspase and/or a change in the capacity to survive executioner caspase activation. To distinguish between these two possibilities, we used cDcp1 staining to assess cell death. If a gene is required for cell survival from executioner caspase activation, we expect RNAi to cause an increase in the percentage of cDcp1^+^ dead cells (cells that die after executioner caspase activation) at the expense of CasExpress^+^ cells (cells that live after executioner caspase activation). By contrast, if knocking down a gene affects apoptosis initiation and/or caspase activity but not the capacity to survive executioner caspase activation, we expect the percentages of CasExpress^+^ cells and of cDcp1^+^ dead cells to change in the same direction (Fig. 4a). However, due to the very low level of cDcp1^+^ cells in control tissues, it is difficult to detect a significant reduction of cDcp1^+^ cells. Thus, in discs with decreased CasExpress activation, if the percentage of cDcp1^+^ cells did not increase, we considered that the knockdown more likely affected caspase activation.

**Fig. 5:**
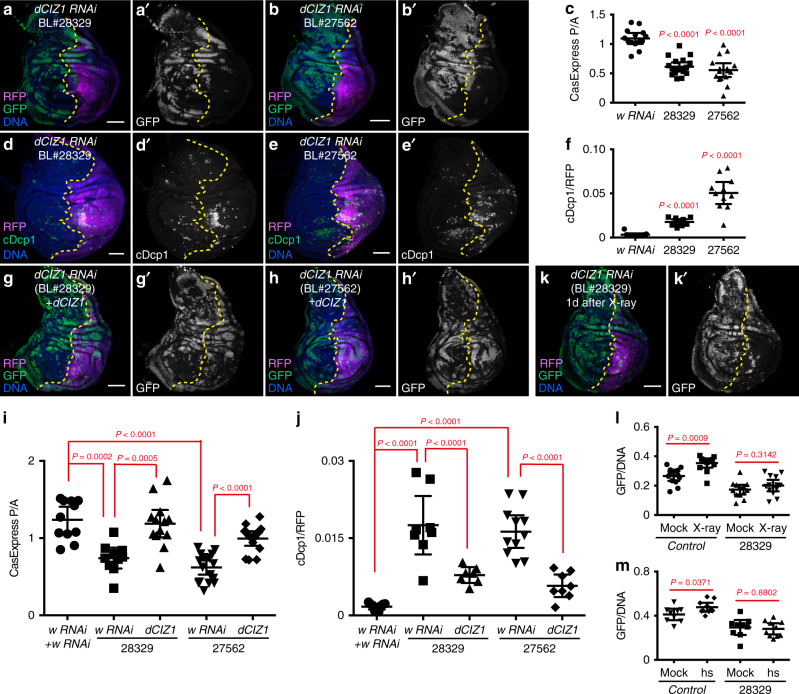
dCIZ1 is essential for survival from executioner caspase activation. (**a**, **b**) Expression of two different RNAi against *dCIZ1* reduced CasExpress activation (GFP). (**c**) Quantification of CasExpress activation in discs expressing *enGal4*, *UAS-RFP*, *L-CasExpress*, *L-trace*, and *w RNAi* (*n* = 14) or *BL#28329* (*n* = 18) or *BL#27562* (*n* = 16). (**d**, **e**) Expression of two different RNAi against *dCIZ1* increased cell death (cDcp1^+^). (**f**) Quantification of cDcp1^+^ area in compartments expressing *w RNAi* (*n* = 11) or *BL#28329* (*n* = 10) or *BL#27562* (*n* = 11) (marked by RFP). (**g**–**i**) Overexpression of *dCIZ1* in *dCIZ1 RNAi* compartment restored CasExpress activation (GFP). *n* = 11 (*w RNAi* + *w RNAi*), 10 (*28329* + *w RNAi*), 13 (*28329* + *dCIZ1*), 17 (*27562* + *w RNAi*), 15 (*27562* + *dCIZ1*). (**j**) Overexpression of *dCIZ1* suppressed cell death induced by *dCIZ1 RNAi*. *n* = 9 (*w RNAi* + *w RNAi*), 8 (*28329* + *w RNAi*), 7 (*28329* + *dCIZ1*), 11 (*27562* + *w RNAi*), 8 (*27562* + *dCIZ1*). (**k**) radiation cannot induce CasExpress activation (GFP) in *dCIZ1 RNAi* compartment. (**l**) Quantification of the percentage of GFP^+^ cells in the anterior compartment (control) and in the *dCIZ1 RNAi* (BL#28329) compartment in mock-treated (mock) or radiated discs (X-ray). *n* = 13 for both groups. (**m**) Heat shock cannot induce CasExpress activation (GFP) in *dCIZ1 RNAi* (BL#28329) compartment. The plot shows quantification of the percentage of GFP^+^ cells in the anterior compartment (control) and in the *dCIZ1 RNAi* compartment in mock-treated or heat-shocked discs. *n* = 9 (mock), 10 (hs). In all images, RFP marks *UAS* transgene expressing region. The yellow dotted lines mark the boundary between the anterior compartment and the posterior compartment. Scale bar is 50 μm. In all plots, n is the number of biological independent samples used for quantification. The data are presented as mean values ± 95% confidence interval. Statistical significance was determined after logarithm transformation using unpaired two-tailed *t*-test (**l**, **m**) or one-way ANOVA (**c**, **f**, **i**, **j**). The Tukey test was used to derive the adjusted *P*-value for multiple comparisons. Source data are provided as a Source Data file.

We screened 102 RNAi lines targeting 84 different genes which, when combined with *en-Gal4*, survived to late third instar (Supplementary data set 1). RNAi against the *w* gene served as a negative control (Fig. 4b, b’). We found 13 genes that, when knocked down, increased both the percentage of CasExpress^+^ cells and the percentage of cDcp1^+^ dead cells, indicating that knocking down these genes led to executioner caspase activation in more cells (Fig. 4a, Supplementary data set 1). (Note that any given cell is typically either CasExpress+ and alive, or cDcp1+ and dead). Some known, general pro-survival and proliferation genes like *thickveins (tkv)*, *STAT92E*, *jub*, *Notch (n)*, appeared in this category (Supplementary Figure S3a–c, Supplementary data set 1). We found knockdown of any of 7 genes reduced the percentage of CasExpress^+^ cells without elevating the percentage of cDcp1^+^ cells (Supplementary data set 1), suggesting knocking down these genes reduced executioner caspase activation frequency within the compartment. One gene in this category is *expanded* (*ex*), a known tumor suppressor. Knocking down *ex* abolished CasExpress^+^ cells but did not induce extra cDcp1^+^ cells (Figure S3d–f), consistent with the known effects of *ex* on expression of apoptosis inhibitors like Diap1 and bantam^30^. Interestingly, we identified 3 genes, *akt1*, *bip2*, and *CG8108* for which RNAi resulted in a reduced percentage of CasExpress^+^ cells (Figs. 4c–d, 5a–c, Supplementary Figure S3g–h) and increased percentage of cDcp1^+^ dead cells (Figs. 4f, g, 5d–f, Supplementary Figure S3i), indicating these genes are required for survival from executioner caspase activation.

### Akt1 is required for survival from stress-induced executioner caspase activation

To test directly whether Akt1 is required for survival following an imposed apoptosis-inducing stress, we heat-shocked *enGal4; UAS-akt1RNAi* larvae and evaluated the effect on L-CasExpress activation one day later. In this genotype, developmental L-CasExpress activation is not blocked before stress, so even unstressed (mock-treated) animals exhibit L-CasExpress activity. Nevertheless, heat shock-induced a significant increase in L-CasExpress activation in the anterior compartment of the wing discs, where *akt1 RNAi* was not expressed (Fig. 4h, h’, i). In contrast, no significant heat-shock-induced increase in L-CasExpress activation was detected in the *akt1 RNAi*-expressing compartments (Fig. 4h, h’, i).

### CG8108, a Drosophila homolog of CIZ1, is essential for survival from executioner caspase activation

Of the 102 RNAi lines we tested for effects on CasExpress activation, two (BL#36665 and BL#28329) were supposed to target the gene *chico*. When expressed with *en-Gal4*, both reduced CasExpress activation (Fig. 5a, a’, c, Supplementary Figure S4a, b). However, expression of BL#28329 dramatically increased the number of cDcp1^+^ cells (Fig. 5d, d’, f) whereas BL#36665 did not (Supplementary Figure S4a”). We thought that BL#28329 might provide a stronger knockdown than BL#36665, so we used qPCR to compare their effects on *chico* mRNA levels. Surprisingly, BL#36665 reduced *chico* mRNA level to one-fourth, whereas BL#28329 had no measurable effect (Figure S4c), indicating BL#28329 did not target *chico*.

To identify the likely target of this RNAi line, we sequenced the hairpin and found that BL#28329 targeted *CG8108*, rather than *chico*, indicating that it was mislabeled in the stock center. We confirmed with qPCR that expression of BL#28329 reduced the mRNA level of *CG8108* by half (Figure S4d).

*CG8108* is an uncharacterized gene encoding a protein homologous to human CDKN1A-interacting zinc finger protein 1 (CIZ1). A second RNAi line against *dCIZ1* (*CG8108*) (BL#27562) caused similar phenotypes as BL#28329, including reduced CasExpress activation (Fig. 5b, b’, c) and increased cDcp1^+^ cells (Fig. 5e, e’, f). These phenotypes were rescued by overexpression of dCIZ1 (Fig. 5g–j). Furthermore, *dCIZ1* knockdown prevented heat-shock- or radiation-induced L-CasExpress activation (Fig. 5k–m). These data together suggest dCIZ1 is essential for survival from executioner caspase activation.

Overexpression of dCIZ1 was not sufficient to protect against *rpr*-induced apoptotic cell death (Supplementary Figure S5a–c). There was no cleaved form of dCIZ1 specific to apoptotic cells (Supplementary Figure S5d). Nor was there a detectable physical interaction between dCIZ1 and Drice or cleaved Drice (Supplementary Figure S5e), suggesting dCIZ1 may not promote survival through direct interaction with Drice.

### dCIZ1 required for survival from executioner caspase activation in tissues with activated PI3K/Akt signaling

**Fig. 6:**
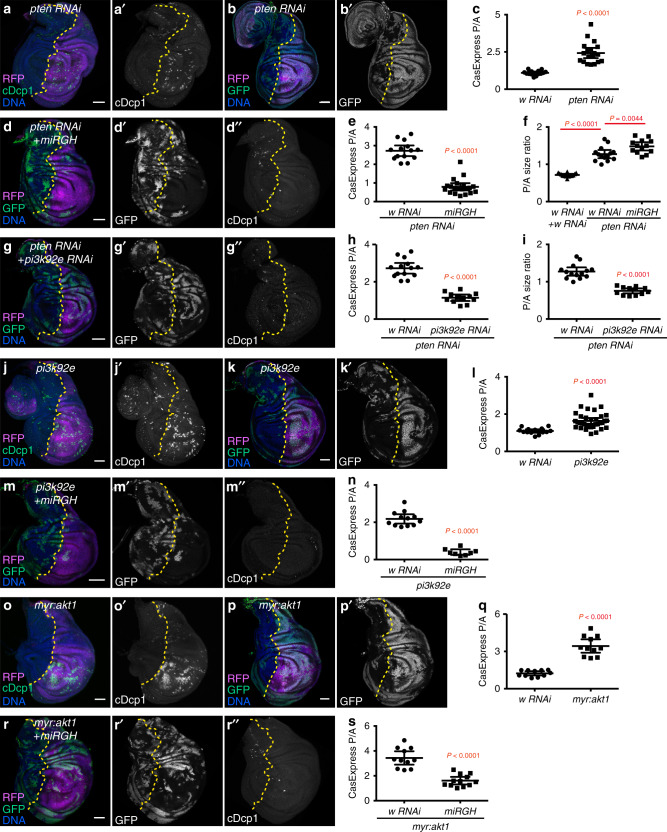
Activation of PI3K-Akt1 signaling induces autonomous apoptotic stress and promotes survival from executioner caspase activation. (**a**–**c**) Knocking down *pten* induced cell death (cDcp1^+^) (**a**) and increased CasExpress activation (GFP) (**b**, **c**). *n* = 14 (*w RNAi*), 19 (*pten RNAi*). (**d**, **e**) Overexpression of *miRGH* in the *pten RNAi* compartment strongly reduced CasExpress activation and eliminated cell death. *n* = 14 (*pten RNAi* + *w RNAi)*, 20 (*pten RNAi* + *miRGH*). (**f**) Knocking down *pten* caused overgrowth of posterior compartment (quantified by the ratio of the posterior compartment size over the anterior compartment size, P/A size ratio), which was further increased by co-overexpression of *miRGH*. *n* = 14 (*w RNAi* + *w RNAi*), *n* = 14 (*pten RNAi* + *w RNAi)*, 13 (*pten RNAi* + *miRGH*). (**g**–**h**) Knocking down *pi3k92e* suppressed the elevated CasExpress activation and cell death caused by *pten RNAi*. *n* = 14 (*pten RNAi* + *w RNAi)*, 12 (*pten RNAi* + *pi3k92e RNAi*). (**i**) Knocking down *pi3k92e* suppressed overgrowth of the *pten RNAi* compartment. *n* = 14 (*pten RNAi* + *w RNAi)*, 12 (*pten RNAi* + *pi3k92e RNAi*). (**j**–**l**) Overexpression of *pi3k92e* increased cell death (cDcp1^+^) (**j**) and CasExpress activation (GFP) (**k**, **l**). *n* = 14 (*w RNAi*), 34 (*pi3k92e*). (**m**, **n**) Overexpression of *miRGH* blocked both CasExpress activation and cell death. *n* = 12 (*pi3k92e* + *w RNAi*), 8 (*pi3k92e* + *miRGH*). (**o**–**q**) Overexpression of *myr:akt1* for 3d increased cell death (cDcp1^+^) (**o**) and CasExpress activation (GFP) (**p**–**q**). *n* = 11 (*w RNAi*), 11 (*myr:akt1*). (**r**, **s**) Overexpression of *miRGH* suppressed *myr:akt1*-induced elevation in CasExpress activation and cell death. *n* = 11 (*myr:akt1* + *w RNAi*), 12 (*myr:akt1* + *miRGH*). Discs used in (**o-s**) contained *Gal80*^*ts*^ to control the expression timing of *myr:akt1*. In all images, RFP marks *UAS* transgene expressing region. The yellow dotted lines mark the boundary between the anterior compartment and the posterior compartment. Scale bar is 50 μm. In all plots, n is the number of biological independent samples used for quantification. The data are presented as mean values + ±95% confidence interval. Statistical significance was determined after logarithm transformation using unpaired two-tailed t-test (all plots other than **f**) or one-way ANOVA (**f**). The Tukey test was used to derive adjusted P-value for multiple comparisons. Source data are provided as a Source Data file.

The phosphinositide-3-kinase (PI3K) pathway (Supplementary Figure S6a) is associated with cell proliferation and survival in many contexts^31^. RNAi screening revealed potent effects—some expected and others not—of multiple components of PI3K pathway on cell death and on CasExpress activation (Supplementary Figure S6b–g, Supplementary data set 1). For example, the phosphatase and tensin homolog (PTEN) is a negative regulator of PI3K signaling and an established tumor suppressor^31^. RNAi against *pten* resulted, unsurprisingly, in compartment overgrowth (Fig. 6a, a’, f). However, *pten RNAi* also resulted in increased apoptotic cell death detected with anti-cDcp1 staining (Fig. 6a, a’), suggesting that tissue overgrowth due to hyperactive PI3K signaling might cause stress and induce apoptosis. Increased CasExpress activation in the *pten RNAi*-expressing compartment (Fig. 6b, c) shows not only increased death but also increased survival following apoptotic caspase activation.

The pattern of increased cDcp1 and CasExpress indicates that *pten* loss increased apoptotic induction of caspase activity (Fig. 4a). To test this idea, we inhibited *rpr*/*hid*/*grim* expression in cells expressing *pten RNAi*. Overexpressing *miRGH* in the wing disc compartment expressing *pten RNAi* reduced CasExpress activation by two-thirds (Fig. 6d, e) and virtually eliminated cDcp1^+^ dead cells (Fig. 6d”). Thus, the executioner caspase activation and cell death within the *pten RNAi* compartment required pro-apoptotic genes. The observation that *miRGH* expression in the *pten RNAi* domain further increased the compartment size by 15% (Fig. 6f), suggests that apoptosis restrained tissue overgrowth to some extent.

**Fig. 7:**
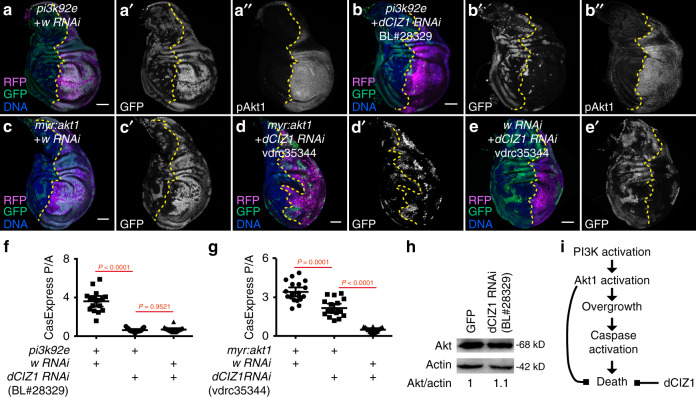
dCIZ1 promotes survival from executioner caspase activation in PI3K- or Akt1-activated tissues. (**a**, **b**) Knocking down *dCIZ1* suppressed CasExpress activation but not Akt1 activation in the compartment expressing *pi3k92e*. *dCIZ1 RNAi* (BL#28329) was used in (**b**). (**c**–**e**) Knocking down *dCIZ1* suppressed *myr:akt1*-induced CasExpress activation. *dCIZ1 RNAi* (vdrc35344) was used in (**c**–**e**), and *Gal80*^*ts*^ was used to limit expression of the transgenes to 3 days. (**f**) Quantification of CasExpress P/A in discs expressing *L-CasExpress*, *L-trace*, *en-Gal4*, *UAS-RFP*, together with *UAS-pi3k92e* and *UAS-w RNAi* (*n* = 17), or *UAS-pi3k92e* and *UAS-dCIZ1 RNAi* (BL#28329) (*n* = 15), or *UAS-dCIZ1 RNAi* (BL#28329) and *UAS-w RNAi* (*n* = 18). (**g**) Quantification of CasExpress P/A in discs expressing *L-CasExpress*, *L-trace*, *en-Gal4*, *UAS-RFP*, *tubGal80*^*ts*^ together with *UAS-myr:akt1* and *UAS-w RNAi* (*n* = 20), or *UAS-myr:akt1* and *UAS-dCIZ1 RNAi* (vdrc35344) (*n* = 18), or *UAS-dCIZ1 RNAi* (vdrc35344) and *UAS-w RNAi* (*n* = 14). (**h**) Expression of *dCIZ1 RNAi* (BL#28329) under *en-Gal4* did not affect dCIZ1 protein level. The numbers below the blots are the relative ratio between the intensity of Akt band and that of actin band. (**i**) The schematic of the role of Akt1 and dCIZ1 in promoting survival from executioner caspase activation in tissues with activated PI3K-Akt1 signaling. In all images, RFP marks *UAS* transgene expressing region. The yellow dotted lines mark the boundary between the anterior compartment and the posterior compartment. Scale bar is 50 μm. In all plots, n is the number of biological independent samples used for quantification. The data are presented as mean values ± 95% confidence interval. Statistical significance was determined after logarithm transformation using one-way ANOVA. The Tukey test was used to derive adjusted P-value for multiple comparisons. Source data are provided as a Source Data file.

To test which effects of *pten RNAi* were consequences of activation of PI3K pathway, we manipulated PI3K signaling in the *pten RNAi* compartment. Knocking down the catalytic subunit of the PI3K complex, *pi3k92e*, together with *pten RNAi* suppressed CasExpress activation, cDcp1^+^ dead cells (Fig. 6g, h), and overgrowth (Fig. 6i). Furthermore, overexpression of *pi3k92e* elevated both cell death (Fig. 6j) and CasExpress activation (Fig. 6k, l). Similar to *pten RNAi*, *pi3k92e*-induced CasExpress activation and cell death was inhibited by overexpression of *miRGH* (Fig. 6m, n). Constitutively activated *akt1* (*myr:akt1*) caused similar effects (Fig. 6o–s). These data suggest that activation of PI3K-Akt1 signaling promotes tissue overgrowth, which results in apoptotic stress in the overgrown compartment, possibly due to overgrowth-induced cell crowding^32^ and/or competition for nutrients or survival factors^33,34^. However, activated Akt1 also promotes survival from executioner caspase activation, which contributes to tissue overgrowth (Fig. 7i). These results underscore the multifaceted effects of PI3K/Akt signaling on multicellular tissues.

The discovery of dCIZ1 as an important regulator of cell survival from caspase activation during normal development raised the question as to whether survival from executioner caspase activation generally requires dCIZ1. We found that CasExpress activation in *pi3k92e*-overexpressing discs (Fig. 7a-a”) was eliminated by dCIZ1 knockdown (b-b” and f), indicating an essential role of dCIZ1 in promoting survival in the context of activated PI3K. Next, we tested the effect of *dCIZ1 RNAi* on *myr:akt1*-induced CasExpress activation. Knocking down *dCIZ1* significantly suppressed *myr:akt1*-induced CasExpress activation (Fig. 7c–e, g). Because both *UAS-myr:akt1* and the two Bloomington *dCIZ1 RNAi* are on the third chromosome, we used another *dCIZ1 RNAi* from Vienna Drosophila Resource Center, vdrc35344, in this experiment. Similar to BL#28329 and BL#27562, expression of vdrc35344 increased the fraction of cDcp^+^ dead cells (Supplementary Figure S7a). The increased cell death in vdrc35344-expressing discs was rescued by co-expressing *dCIZ1* (Supplementary Figure S7b), suggesting vdrc35344 targets on *dCIZ1*.

It has been reported that knocking down *CIZ1* in human prostate cancer cells reduced Akt expression^35^. However, neither the Akt level (Fig. 7h) nor the PI3K-induced Akt phosphorylation was affected in *dCIZ1 RNAi* discs (compare Fig. 7a”, b”). These data together are consistent with a function for dCIZ1 downstream of or in parallel to Akt1 (Fig. 7i), although we cannot formally exclude the possibility that dCIZ1 may act upstream of Akt1.

### Akt1 and dCIZ1 promote survival of cells with oncogenic Ras^V12^-induced executioner caspase activation

**Fig. 8:**
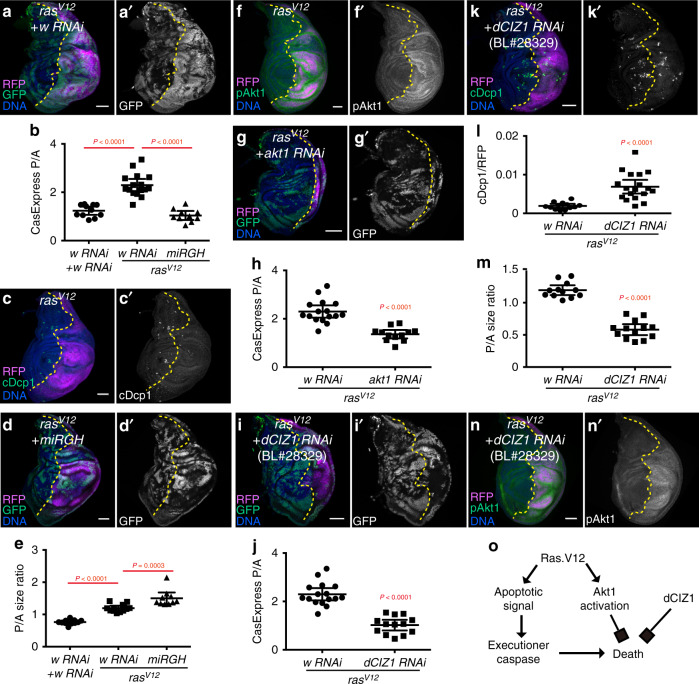
Akt1 and dCIZ1 promote survival from apoptotic executioner caspase activation in tissues with oncogenic Ras^V12^. (**a**) Expression of *ras*^*V12*^ induced CasExpress activation (GFP). (**b**) Quantification of CasExpress activation in discs expressing *w RNAi* + *w RNAi* (*n* = 11), *ras*^*V12*^ + *w RNAi* (*n* = 16), *ras*^*V12*^ + *miRGH* (*n* = 10) under *en-Gal4*. (**c**) Expression of *ras*^*V12*^ induced little cell death. (**d**) *miRGH* inhibited CasExpress activation in *ras*^*V12*^-expressing compartment. (**e**) Expression of *ras*^*V12*^ induced overgrowth of the compartment, which was further increased by overexpression of *miRGH*. *n* = 11 (*w RNAi* + *w RNAi*), 12 (*ras*^*V12*^ + *w RNAi*), 10 (*ras*^*V12*^ + *miRGH*). (**f**) Expression of *ras*^*V12*^ activated Akt1. (**g**, **h**) Knocking down *akt1* suppressed *ras*^*V12*^-induced CasExpress activation. *n* = 16 (*ras*^*V12*^ + *w RNAi*), 12 (*ras*^*V12*^ + *akt1 RNAi*). (**i**, **j**) Knocking down *dCIZ1* blocked CasExpress activation in *ras*^*V12*^-expressing compartment. *n* = 16 (*ras*^*V12*^ + *w RNAi*), 13 (*ras*^*V12*^ + *dCIZ1 RNAi*). (**k**, **l**) Knocking down *dCIZ1* induced cell death (cDcp1^+^) in *ras*^*V12*^-expressing compartment. *n* = 13 (*ras*^*V12*^ + *w RNAi*), 18 (*ras*^*V12*^ + *dCIZ1 RNAi* BL#28329). (**m**) Knocking down *dCIZ1* suppressed *ras*^*V12*^-induced overgrowth. *n* = 12 (*ras*^*V12*^ + *w RNAi*), 13 (*ras*^*V12*^ + *dCIZ1 RNAi* BL#28329). (**n**) Expression of *dCIZ1 RNAi* (BL#28329) did not affect *ras*^*V12*^-induced Akt1 activation. (**o**) The schematic of the role of Akt1 and dCIZ1 in tissues with oncogenic Ras^V12^. *Gal80*^*ts*^ was used in the experiments in this figure to control the timing of *ras*^*V12*^ expression. In all images, RFP marks *UAS* transgene expressing region. The yellow dotted lines mark the boundary between the anterior compartment and the posterior compartment. Scale bar is 50 μm. In all plots, *n* is the number of biological independent samples used for quantification. The data are presented as mean values ± 95% confidence interval. Statistical significance was determined after logarithm transformation using unpaired two-tailed *t*-test (**h**, **j**, **l**, **m**) or one-way ANOVA (**b**, **e**). The Tukey test was used to derive adjusted *P*-value for multiple comparisons. Source data are provided as a Source Data file.

Hyperactivation of PI3K-Akt1 signaling occurs in a large variety of human cancers. The human *PIK3CA* gene, which encodes the catalytic subunit of the PI3K complex, is the second most frequently mutated oncogene, and *PTEN* is one of the most frequently mutated tumor suppressor genes^31^. The discovery of induced apoptotic stress and survival from executioner caspase activation in tissues with activated PI3K-Akt signals led us to test whether expression of another common oncogenic mutation, Ras^V12^, would also induce autonomous apoptotic stress and subsequent survival from executioner caspase activation. Expression of *ras*^*V12*^ with *en-Gal4* was lethal, so we used Gal80^ts^ to limit the expression to three days. Expression of oncogenic *ras*^*V12*^ in the posterior compartment led to a more than two-fold increase in the fraction of cells with CasExpress activation (Fig. 8a, b). Strikingly, few if any cDcp1^+^ cells were detectable (Fig. 8c), indicating that most cells that activated executioner caspases survived. Co-expressing *miRGH* to inhibit apoptosis induction in the *ras*^*V12*^-expressing compartment suppressed the elevated CasExpress (Fig. 8b, d). We conclude that *ras*^*V12*^-induced CasExpress activation requires apoptosis induction.

Mild activation of executioner caspases in *scrib*^*−*^ *ras*^*V12*^ clones in eye discs can promote neoplastic growth^36^. However, in *ras*^*V12*^-expressing disc cells, overexpression of *miRGH* caused a small increase rather than reduction in the compartment size (Fig. 8e). Therefore, apoptosis restrains the overgrowth of tissues with oncogenic mutations including both *ras*^*V12*^ and, as shown above, PI3K. Counteracting this growth-inhibiting apoptosis, Ras^V12^ also promotes survival from executioner caspase activation, which eventually contributes to tissue overgrowth. Promoting survival from caspase activation due to apoptotic stress represents a previously unknown function for activated Ras.

Consistent with a previous report that PI3K signaling is activated in *ras*^*V12*^-expressing cells^37^, we detected increased active Akt1 (phospho-Akt1) in the *ras*^*V12*^-expressing compartment (Fig. 8f). Knocking down *akt1* in the *ras*^*V12*^-expressing compartment blocked the induced CasExpress activation (Fig. 8g–h), suggesting Ras^V12^ promotes survival from executioner caspase activation through Akt1. Knocking down *dCIZ1* in *ras*^*V12*^-overexpressing discs also suppressed CasExpress activation (Fig. 8i, j), induced apoptotic cell death (Fig. 8k, l), and suppressed Ras^V12^-induced tissue overgrowth (Fig. 8m). Compartments co-expressing *dCIZ1 RNAi* and *ras*^*V12*^ showed high Akt1 activity (Fig. 8n), again indicating dCIZ1 may function downstream of or in parallel to Akt1.

Based on these data, we propose that during Ras^V12^-induced oncogenic transformation, competing death and survival signals are activated. Apoptosis is initiated and executioner caspases are activated, either as a homeostatic mechanism or as a consequence of nutrient-deprivation, hypoxia, or mechanical stress. In any case, dCIZ1 and activated Akt1 in the transformed cells promote survival of stressed cells, thereby contributing to oncogenic overgrowth (Fig. 8o).

## Discussion

Here we show that cells can survive executioner caspase activation induced by apoptotic signals during normal development, following imposed apoptosis-inducing stresses such as heat shock, radiation, and *rpr* overexpression, and in response to oncogene activation. We show that cells can survive executioner caspase activation and contribute to tissue regeneration following severe imposed stresses. The wing disc is a highly regenerative tissue. It can regenerate even after half of the cells die^38^. A widely-acknowledged mechanism underlying wing disc regeneration is apoptosis-induced proliferation, in which apoptotic cells emit mitogenic signals to promote proliferation of surrounding healthy cells^18,39–41^. The initiator caspase Dronc is required for mitogenic signal production in apoptotic cells^42^. Apoptosis and executioner caspases also play indispensable roles in regeneration in other model organisms like planaria, hydra, and mice^43–47^. For example, loss of either caspase-3 or -7 impairs skin wound repair and liver regeneration in mice^44^. In the classic apoptosis-induced regeneration model, cells initiate apoptosis, activate executioner caspases, and die; the regenerated epithelium originates from healthy cells that never activate executioner caspase. Here we report that even cells that activate executioner caspases can survive and participate in regeneration.

The fraction of the regenerated epithelium that originates from cells that survive apoptotic caspase activation varies depending on the conditions. In irradiated larvae, the majority of cells in the recovered disc are progeny of cells that have experienced executioner caspase activation (Fig. 1). In contrast, when discs regenerate after local and transient *rpr* overexpression, fewer CasExpress^+^ cells contribute to recovery (Fig. 2). The difference could be due to different rates or levels of caspase activation^20^ and/or to competition from unstressed cells^48,49^. In the radiation experiments, all cells were stressed whereas *rpr* overexpression was limited to a small fraction of the disc, so there was a large pool of unstressed cells nearby. These findings suggest that salvaging cells from executioner caspase activation may be particularly important for recovery from a transient apoptosis-inducing stress that affects an entire organ or organism, such as systemic chemotherapy.

One hallmark of cancer is the capacity to evade cell death. An apparent paradox is that caspase activity is prevalent in gastric and breast cancers, melanoma, and lymphoma^50–52^, amongst others. In recent years, apoptosis and caspases have been shown to promote tumor growth, recurrence, and metastasis both autonomously and non-autonomously^36,53–57^. After radiation or chemotherapeutic drug treatment, caspase 3-dependent secretion of growth factors in apoptotic tumor cells can trigger repopulation of non-apoptotic tumor cells and angiogenesis^54–56,58^. Apoptotic lymphoma cells can modify the tumor microenvironment and activate tumor-associated macrophages to promote tumor progression^57^.

In addition to these paracrine effects on tumor progression, executioner caspase activity can also autonomously drive tumor growth and metastasis in Drosophila^36^ and mouse models^53^. In this study, we found that PI3K activation and oncogenic Ras^V12^ mutation, two of the most frequent molecular alterations in tumors, can promote cell survival from apoptotic executioner caspase activation. Our observation that inhibition of survival from executioner caspase activation in *ras*^*V12*^-expressing tissues suppressed overgrowth demonstrates that recovery from apoptotic caspase activation represents a previously unrecognized mechanism by which Ras^V12^ promotes survival.

We identified Akt1 as an essential driver for survival from executioner caspase activation. As a pro-survival protein, Akt is well known to inhibit apoptosis upstream of caspase activation and mitochondrial permeabilization^59,60^. Our work reveals the capacity of Akt1 to promote cell survival after executioner caspase activation in response to apoptotic stresses, consistent with one study that shows that Akt can inhibit apoptosis even after mitochondrial outer membrane permeabilization, at least in one cell type^61^. Khalil et al.^62^ found that exposure of multiple cell types and tissues to apoptosis-inducing radiation or chemicals results in stress-induced Akt activation that is dependent upon caspase 3 induction of apoptosis and necessary for tissue repair and animal survival. Though they did not show Akt was activated in the very same cells that activated caspase 3, since they did not have a CasExpress-like reporter, it seems likely that they detected Akt-mediated survival after executioner caspase activation. These, together with our data, suggest the regulation of survival from executioner caspase activation may be conserved from flies to mammals.

It is exciting then, that in addition to Ras and PI3K/Akt1, the fly screen revealed the importance of CG8108 (dCIZ1). Though there are no published functional studies on dCIZ1, it was identified in the interactome of stress-activated MAP kinases^63^. Remarkably, it also emerged in a PI3K interactome^64^. The human homolog of dCIZ1 has many attributes consistent with a determinant that promotes survival in the face of tissue damage. For example, CIZ1 binds and inactivates the senescence-promoting protein p21^Cip1/Waf1^^65^, thus antagonizing DNA-damage-induced proliferation arrest. It has reported roles in DNA replication^66^, cell cycle progression^67^, transcription^68–70^, and epigenetic regulation^71^. CIZ1 can promote tumor growth and metastasis^70,72–74^ and protect cells from radiation-induced DNA damage and apoptosis^75^. *ciz1*^*−/−*^ mice develop neurodegenerative phenotypes during aging^75^; whereas overexpression of CIZ1 in cardiomyocytes protects the heart from ischemia-reperfusion injury^76^, a physiological condition in which cells survive caspase-3 activation^9^. CIZ1 is also overexpressed in many solid tumors^67,77^. All these studies suggest the role of CIZ1 in promoting survival from stress-induced executioner caspase activation may be conserved from Drosophila to mammals. Activation of PI3K-Akt signaling and oncogenic Ras are common in tumors. The fact that loss of dCIZ1 blocked survival in tissues with high level of PI3K-Akt1 activity or Ras^V12^, suggests it would be an excellent therapeutic target for cancer treatment. The discovery of dCIZ1 as a general regulator of survival from executioner caspase activation in development, regeneration, and oncogene-driven proliferation thus represents a significant advance.

## Methods

### Fly stocks

To construct the L-CasExpress sensor, DNA encoding mCD8, and amino acids 2-147 of Diap1 (mCD8-DQVD) was cloned from the previously reported CasExpress sensor^11^. DNA encoding lexA::p65 fusion protein was cloned from pBPLexA::p65Uw (Addgene#26231). The *mCD8-DQVD* and *lexA::p65* fragments were then ligated into pattB-Ubi vector^11^ using In-Fusion Cloning Kit (Clontech). The construct was injected into attP2 site by BestGene Inc.

To make UAS-dCIZ1:3Xflag transgenic flies, we cloned the cDNA of dCIZ1 using clone LD27033 (Drosophila Genomics Resource Center) as the template and put it into the pJFRC7-20XUAS vector (made from Addgene#26220). The construct was injected into attP40 site by BestGene Inc.

All the RNAi stocks used in the RNAi screening are listed in Supplementary data set 1. The other fly stocks used in this study are listed in Supplementary Table S1. The *L-trace* flies were made through recombination of *lexO-flp* (II) and *FRT-STOP-FRT-GFP* (II).

The genotypes of the samples shown in the figures are listed in Supplementary Table S2.

### Stress and recovery assays

For all stress and recovery experiments including heat shock, radiation, and transient *rpr* overexpression, embryos were collected in an 8 h window at 25 °C, and the embryo collection plates were then transferred to 18 °C. 2 days after embryo collection, larvae were collected into food vials with additional dry yeast at 50 larvae per vial. For heat shock and radiation, larvae were heat shock at 37 °C for 2h or irradiated with 10 Gy X-ray at 0.3069 Gy/min on day 7 after egg-laying, then transferred to 29 °C for 1d, followed by dissection or an additional 3 days at 18 °C. For transient *rpr* overexpression, on day 7 after egg-laying, larvae were shifted to 29 °C for 1d, then the larvae were either dissected or shifted back to 18 °C for recovery.

### RNAi screening

CasExpress is activated in sperm due to the essential role of executioner caspase in sperm differentiation^25^. Thus CasExpress and L-trace need to be kept in separate stocks. We first made *en-Gal4 UAS-RFP/CyOGFP; L-CasExpress/TM6B* stock and *en-Gal4 UAS-RFP L-trace/CyO* stock for RNAi screening. Each *UAS-RNAi* on the third chromosome was crossed into *L-trace* background, and the *L-trace/CyOGFP; UAS-RNAi/TM6B* flies were then crossed to *en-Gal4 UAS-RFP/CyOGFP; L-CasExpress/TM6B* flies to get the *en-Gal4 UAS-RFP/L-trace; L-CasExpress/UAS-RNAi* larvae. Each *UAS-RNAi* on the second chromosome was crossed into *L-CasExpress* background, and the *UAS-RNAi/CyOGFP; L-CasExpress/TM6B* flies were then crossed to *en-Gal4 UAS-RFP L-trace/CyO* flies to get *en-Gal4 UAS-RFP L-trace/UAS-RNAi; L-CasExpress/+* larvae. For genes like *akt1*, which when knocked down at 25 °C causes lethality, we used Gal80^ts^ to limit the knock-down to 3 days. The wing disc dissection, imaging, and quantification procedures are described in detail in the following sections. RNAi that caused a statistically significant change higher than 20% on CasExpress P/A were considered as candidates for regulators. Among the candidates, only those with cDcp1^+^ cells changed in the direction opposite to change in CasExpress P/A were considered as regulators for survival from executioner caspase activation.

### Immunostaining and imaging

Larvae at mid or late third instar were dissected and fixed in 4% paraformaldehyde (Electron Microscope Sciences) for 20 min at room temperature. After fixation, samples were rinsed with PBS twice and washed with PBS + 0.2% Trition X-100 (Sigma) (0.2% PBT) for 20 min. Samples were then blocked with 5% goat serum in 0.2% PBT for 30 min, followed by overnight incubation with primary antibody solution at 4 °C. Next day, the samples were washed with 0.2% PBT three times, and incubated with secondary antibody and Hoechst solution (Molecular Probes) for 1 h at room temperature. Samples were then washed three times with 0.2% PBT and mounted on slides using Vectashield mounting medium (Vector Laboratories). The primary and secondary antibodies used in the immunostaining are listed in Supplementary Table S3. Images were taken using Zeiss LSM 780 confocal microscope and Zeiss LSM 880 confocal microscope. The software used in image capture is Zen Black (version 14.0, Zeiss).

### Image analysis and quantification

All the image analysis and quantification were done using ImageJ (version 2.0). To evaluate the effect of transgene on CasExpress activation, the GFP^+^ nuclei area and total nuclei area in both the posterior compartment (P) and the anterior compartment (A) was measured. Then the percentage of GFP^+^ nuclei in each compartment was calculated, and CasExpress P/A ratio was calculated. To quantify cell death, cDcp1^+^ area and RFP area were measured and the ratio between them was calculated.

### RNA extraction, reverse transcription, and qPCR

For each genotype, about 50 late third instar wing discs were dissected in PBS, and immediately transferred to TRIZOL (ThermoFisher Scientific). The total RNA was extracted using standard TRIZOL RNA extraction protocol. Turbo DNase (ThermoFisher Scientific) was used to remove genomic DNA contamination. Reverse transcription was done using SuperScript III First-Strand Synthesis System (ThermoFisher Scientific). qPCR was done using SsoAdvanced Universal SYBR Green Supermix (Bio-rad) on Bio-rad CFX96 real-time PCR detection system. The data were collected using Bio-rad CFX Manager software (version 3.1, Bio-rad). The primers used for qPCR were listed in Supplementary Table S4.

### Transfection, protein sample preparation, co-immunoprecipitation, and western blotting

Drosophila S2 cells (Thermo Fisher Scientific) were cultured in Schneider’s Drosophila medium (Thermo Fisher Scientific) supplemented with 10% fetal bovine serum (Sigma) and 100U/ml Penicillin-Streptomycin (Thermo Fisher Scientific). One day before transfection, S2 cells were seeded in 6-well plates. pMT-Gal4 plasmid and pJFRC-20XUAS-dCIZ1:3Xflag plasmid were transfected into S2 cells using Effectene Transfection Reagent (Qiagen) following the manual. Next day, 500 μM CuSO_4_ was added to the cells to induce Gal4 expression. After one-day induction, cells were collected and lysed in RIPA buffer (for western blotting) or TBS with 1% Trition X-100 (for co-immunoprecipitation) supplemented with 1 mM PMSF. Co-immunoprecipitation was done using anti-FLAG M2 magnetic beads (Sigma). The protein samples were applied to 10% SDS-PAGE. The primary antibodies used in western blotting are listed in Supplementary Table S3. The original scans of all blots were in Source Data file.

### Statistics and reproducibility

All the statistical analyses were done using Prism 6 software (GraphPad). Statistical significance was determined after logarithm transformation using unpaired two-tailed t-test for two-sample comparison or one-way ANOVA for multiple-sample comparison. The Tukey test was used to derive adjusted *P*-value for multiple comparisons. All experiments were independently repeated at least three times to ensure reproducibility, and the representative images are shown in the figures.

### Reporting summary

Further information on research design is available in the Nature Research Reporting Summary linked to this article.

## Supplementary information

Supplementary Information

Supplementary Data 1

Reporting Summary

## Source data

Source Data

## Data Availability

All the raw data supporting the findings of this study are available from the corresponding authors upon request. Source data are provided with this paper.
