# Author Correction: Akt1 and dCIZ1 promote cell survival from apoptotic caspase activation during regeneration and oncogenic overgrowth

**DOI:** 10.1038/s41467-025-60562-2

**Published:** 2025-06-03

**Authors:** Gongping Sun, Xun Ding, Yewubdar Argaw, Xiaoran Guo, Denise J. Montell

**Affiliations:** 1 The Key Laboratory of Experimental Teratology, Ministry of Education and Department of Anatomy and Histoembryology, School of Basic Medical Sciences, Cheeloo College of Medicine, Shandong University, Jinan, Shandong China; 2 Molecular, Cellular, and Developmental Biology Department, University of California, Santa Barbara, CA 93106 USA

Correction to: *Nature Communications* 10.1038/s41467-020-19068-2, published online 12 November 2020

In this article the author’s name Xun Ding was incorrectly written as Xun Austin Ding. The original article has been corrected.

