# Supplementary material for: Akt1 and dCIZ1 promote cell survival from apoptotic caspase activation during regeneration and oncogenic overgrowth
Source: Nat Commun. 2020 Nov 12;11:5726. doi: 10.1038/s41467-020-19068-2 (PMC7664998; doi:10.1038/s41467-020-19068-2)
Supplement: Supplementary file 1 — Supplementary Information [file 41467_2020_19068_MOESM1_ESM.pdf]

Supplementary information

Akt1 and dCIZ1 promote cell survival from apoptotic caspase activation during regeneration and oncogenic overgrowth

Sun et al.

This PDF file contains the following contents:

Supplementary figure S1-S7 and their legends
The legend for Supplementary data set 1
Supplementary table S1-S4
Supplementary reference

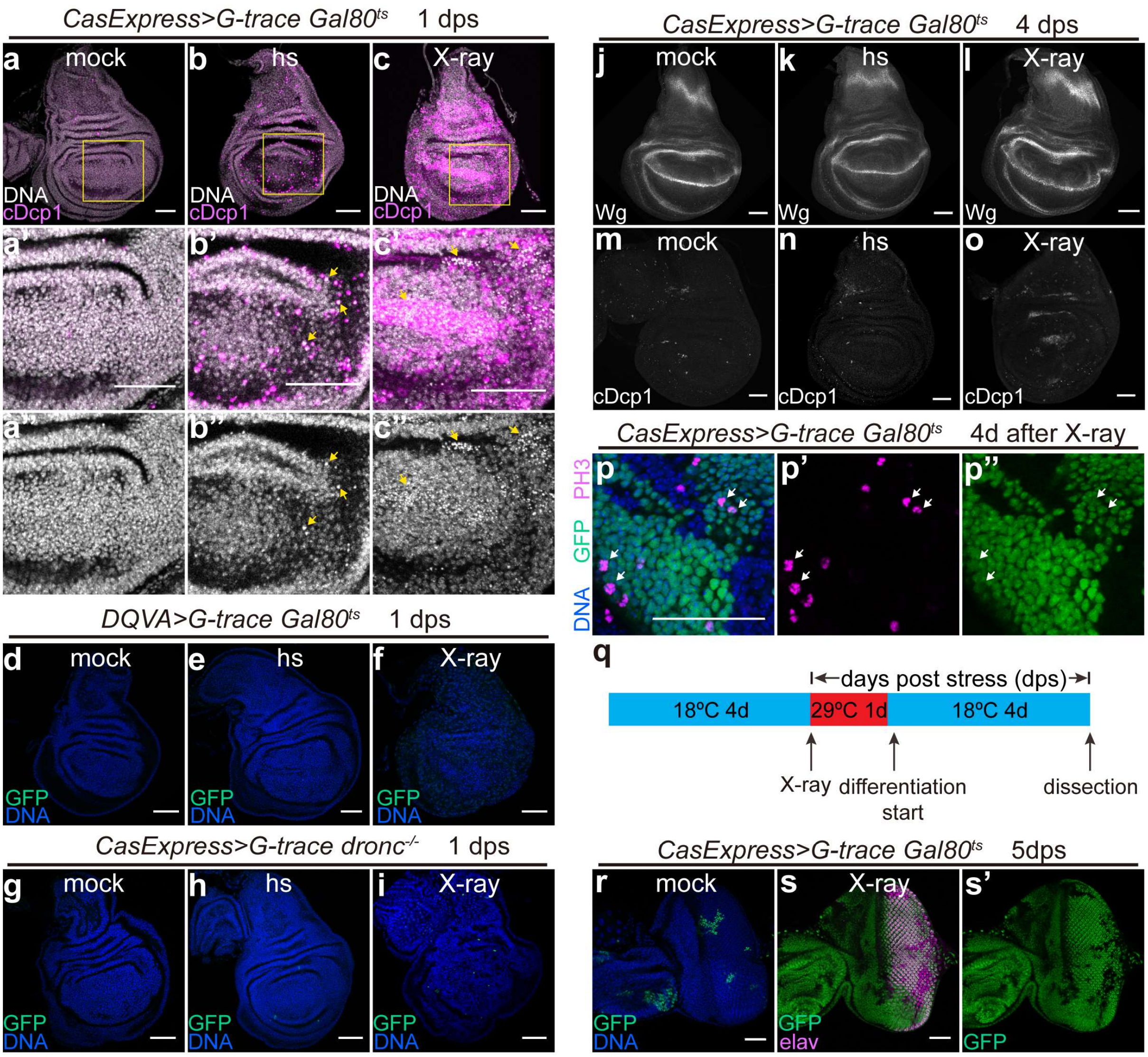

Supplementary figure S1. Tissue damage and regeneration after heat shock or radiation.

a-c) cDcp1 and DNA staining on discs after mock treatment (a), heat shock (b) or X-ray radiation (c) and 1d at 29°C showed that the stresses induced intensive apoptotic cell death. a'-c'') are magnified images of the yellow rectangular area in a-c). Yellow arrows point to several examples of dead cells. d-f) Discs bearing caspase-insensitive sensor DQVA did not give any GFP$^+$ cells after mock treatment (d), heat shock (e) or radiation (f). g-i) CasExpress was not activated by mock treatment (g), heat shock (h), or radiation (i) in *dronc* mutant discs. j-o) Wg staining (j-l) and cDcp1 staining (m-o) on discs after mock treatment (j&m), heat shock (k&n) or radiation (l&o), 1d at 29°C and 3d at 18°C showed that at 4d after stress

the discs restored normal morphology and most of the dead cells were cleared. p) PH3 staining of discs after 4d recovery after X-ray radiation showed $CasExpress^+$ (GFP^+) cells can proliferate. White arrows point to the examples of colocalized PH3 staining and GFP. q) A schematic of the experimental design for results shown in r) and s). r-s) Eye-antennial discs of larvae bearing *CasExpress*, *G-trace* and $Gal80^{ts}$ after mock (r) or X-ray (s), 1d at 29ºC and 4d at 18ºC. Elav staining in s) marks the differentiated neurons. In all images scale bar is 50μm.

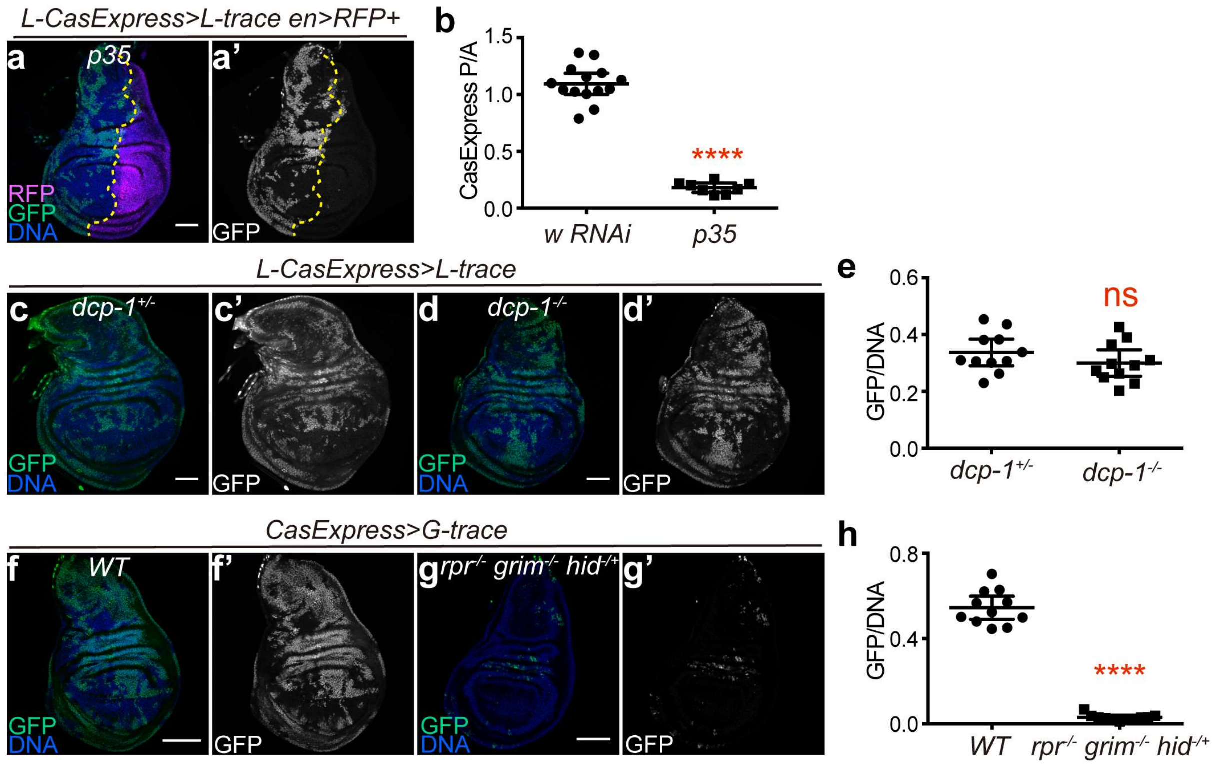

Supplementary figure S2. CasExpress activation during development requires *rpr*, *hid*, *grim* but not *dcp-1*.

a-b) Overexpression of p35 abolished CasExpress activation. In a), RFP marks *UAS* transgene expressing region. The yellow dotted lines mark the boundary between the anterior compartment and the posterior compartment. In b), n=14 (*w RNAi*), 8 (*p35*). c-e) Mutation in *dcp-1* did not affect CasExpress activation (GFP). In e) n=11 for both genotypes. f-h) CasExpress activation was largely reduced in *rpr*$^{-/-}$ *grim*$^{-/-}$ *hid*$^{-/+}$ discs (g) compared to that in wild type (*WT*) discs (f). In h) n=14 (*WT*), 11 (*rpr*$^{-/-}$ *grim*$^{-/-}$ *hid*$^{-/+}$). n is the number of biological independent samples used for quantification. The data are presented as mean values +/- 95% confidence interval. Statistical significance was determined after logarithm transformation using unpaired two-tailed t-test. ns: no statistical significance. ****: P<0.0001. Source data are provided as a Source Data file. In all images, scale bar is 50μm.

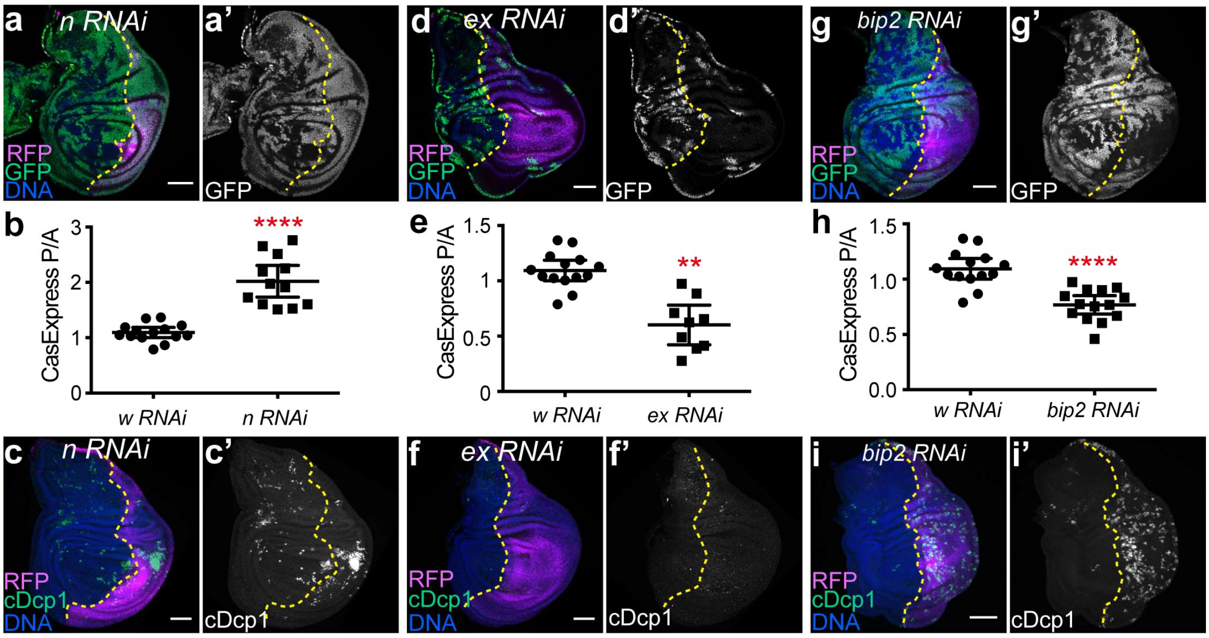

Supplementary figure S3. Examples of genes that regulate survival before or from executioner caspase activation.

a-c) Knocking down *notch* (*n*) increased CasExpress activation (GFP) (a-b) and cell death (cDcp1$^+$) (c). In b), n=14 (*w RNAi*), 12 (*n RNAi*). d-f) Knocking down *expanded* (*ex*) reduced CasExpress activation (d-e) without inducing any cell death (f). n=14 (*w RNAi*), 9 (*ex RNAi*). g-i) Knocking down *bip2* reduced CasExpress activation (g-h) while increased cell death (i). n=14 for both genotypes. In all images, RFP marks *UAS* transgene expressing region. The yellow dotted lines mark the boundary between the anterior compartment and the posterior compartment. Scale bar is 50μm. In all plots, n is the number of biological independent samples used for quantification. The data are presented as mean values +/- 95% confidence interval. Statistical significance was determined after logarithm transformation using unpaired two-tailed t-test. **: P=0.0011. ****: P<0.0001. Source data are provided as a Source Data file.

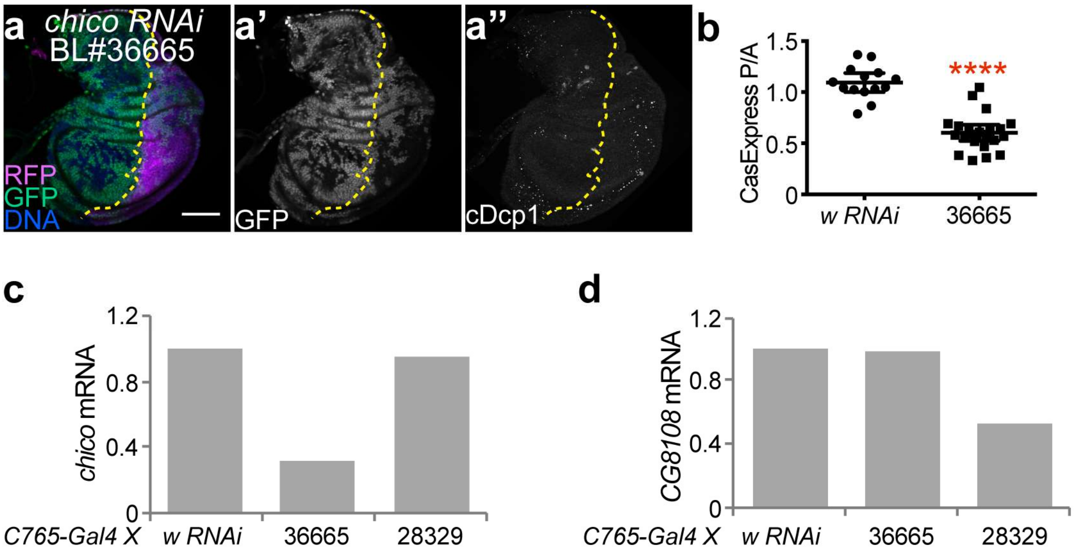

Supplementary figure S4. BL#36665 and BL#28329 target *chico* and *CG8108*, respectively.

a-b) Expression of *chico RNAi* (BL#36665) reduced CasExpress activation (GFP) and had no effect on cell death. n=14 (*w RNAi*), 22 (BL#36665). n is the number of biological independent samples used for quantification. The data in (b) are presented as mean values +/- 95% confidence interval. Statistical significance was determined after logarithm transformation using unpaired two-tailed t-test. ****: P<0.0001. Source data are provided as a Source Data file. Scale bar in (a) is 50μm. c-d) The relative mRNA level of *chico* (c) and *CG8108* (d) in wing discs expressing *C765-Gal4* and *w RNAi*, BL#36665, or BL#28329.

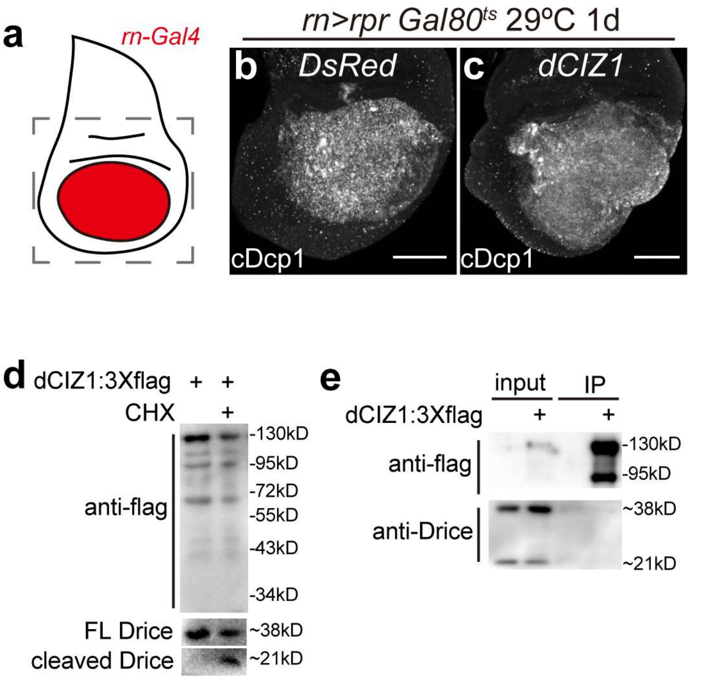

Supplementary figure S5. dCIZ1 does not directly interact with Drice.

a) Schematic shows the *rn-Gal4* expressing region (red). The grey dash rectangular marks the area showed in (b) and (c). b-c) cDcp1 staining in discs of the indicated genotypes after 1d induction of transgene expression. Scale bar is 50µm. d) Western blots of dCIZ1:3Xflag, full length (FL) Drice and cleaved Drice in control cells and apoptotic cells (treated with 20µg/ml CHX for 5hrs). e) Co-immunoprecipitation (Co-IP) of dCIZ1:3Xflag. Input was loaded 1/100 of the samples used in Co-IP.

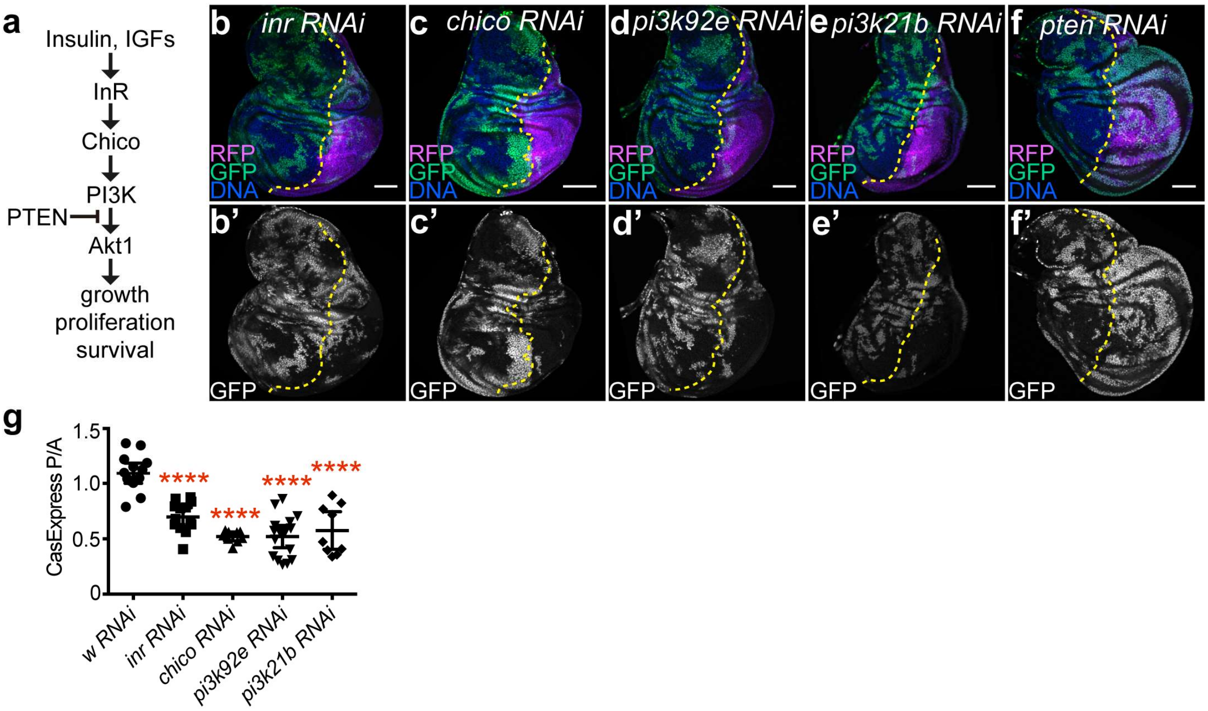

Supplementary figure S6. Interference of insulin receptor-PI3K signaling affects CasExpress activation.

a) The schematic of insulin receptor (InR) -PI3K signaling. b-e) Knocking down *inr* (b), *chico* (c), *pi3k92e* (d) or *pi3k21b* (e) reduced CasExpress activation. f) Knocking down *pten* increased CasExpress activation. g) Quantification of CasExpress activation in b-e). n=14 (*w RNAi*), 13 (*inr RNAi*), 9 (*chico RNAi*), 16 (*pi3k92e RNAi*), 9 (*pi3k21b RNAi*). n is the number of biological independent samples used for quantification. The data are presented as mean values +/- 95% confidence interval. Statistical significance was determined after logarithm transformation using one-way ANOVA. The Tukey test was used to derive adjusted P value for multiple comparisons. ****: P<0.0001. Source data are provided as a Source Data file.

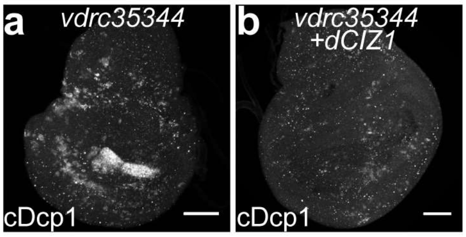

Supplementary figure S7. Expression of *dCIZ1 RNAi* (vdrc35344) resulted in dramatic induction of apoptotic cell death which was suppressed by co-expression of *dCIZ1*.
cDcp1 staining in discs expressing *dCIZ1 RNAi* (vdrc35344) (a) or *dCIZ1 RNAi* (vdrc35344) together with *dCIZ1* (b) under *hh-Gal4*. Scale bar is 50μm.

Supplementary data set 1. Summary of RNAi screening

All RNAi stocks used in the screening are listed in the "all RNAi screened" tab. The effect on CasExpress P/A and cell death (cDcp1^{+}) of the RNAi lines that changed CasExpress activation was listed in "increased CasExpress" and "decreased CasExpress" tabs. Genes that regulates survival from executioner caspase activation were highlighted in red. Statistical significance was determined after logarithm transformation using unpaired two-tailed t-test.

Supplementary table S1. List of fly stocks

Drosophila line	Source
lexO-flp (II)	Bloomington Drosophia Stock Center (BDSC) #55820
FRT-STOP-FRT-GFP (II)	BDSC #32250
G-trace (II)	BDSC #28280
tub-Gal80^{ts} (III)	BDSC #7018
w RNAi (III)	BDSC #33623
en-Gal4 UAS-RFP (II)	BDSC #30557
en-Gal4 (II)	BDSC #30564
dronc RNAi (III)	BDSC #32963
drice RNAi (II)	Vienna Drosophila Resource Center (VDRC) #28065
dCIZ1 RNAi (II)	VDRC #35344
dcp-1^{prev1}	BDSC #63814
dronc^{I29}	gift from K. Irvine[1]
bsk RNAi (II)	BDSC #57035
UAS-puc (III)	gift from A. Martinez-Arias[2]
UAS-p35 (III)	BDSC #5073
UAS-miRGH (II)	gift from D. Bilder[3]
TRE:DsRed (II)	BDSC #59012
MM2 (III)	gift from K. White[4]
H99 (III)	BDSC #1576
salE/Pv-LHG tubGal80^{ts} (III)	gift from F. Serras[5]
lexO-rpr (III)	gift from F. Serras[5]
lexO-mCD8:GFP (II)	BDSC #32205
UAS-pi3k92e (II)	BDSC #8286
UAS-pi3k92e (III)	BDSC #8287
UAS-myr:akt1 (III)	BDSC #50758
UAS-akt1 RNAi (III)	BDSC #36615
UAS-ras^{V12} (II)	BDSC #64196
UAS-ras^{V12} (III)	BDSC #64195
rn-Gal4 UAS-rpr Gal80^{ts}/TM6BGal80	gift from R. Smith-Bolton[6]

Supplementary table S2. Genotypes of the samples used in the figures

Panel #	genotype
Fig 1d-f	*CasExpress/G-trace; tubGal80^{ts}/+*
Fig 1g-i	*CasExpress tubGal80^{ts}/G-trace; H99/MM2*
Fig 1j-l	*CasExpress/G-trace; tubGal80^{ts}/+*
Fig 2b	*lexO-mCD8:GFP/+; salE/Pv-LHG tubGal80^{ts}/lexO-rpr*
Fig 2d	*L-trace/+; salE/Pv-LHG tubGal80^{ts}/+*
Fig 2e	*L-trace/+; salE/Pv-LHG tubGal80^{ts}/lexO-rpr*
Fig 2g & h	*CasExpress/G-trace; +/lexO-rpr*
Fig 2i-k	*CasExpress/G-trace; salE/Pv-LHG tubGal80^{ts}/lexO-rpr*
Fig 3b	*L-trace/+; L-CasExpress/+*
Fig 3c	*enGal4 UAS-RFP/L-trace; UAS-w RNAi/L-CasExpress*
Fig 3d	*enGal4 UAS-RFP/L-trace; UAS-dronc RNAi/L-CasExpress*
Fig 3f	*enGal4 UAS-RFP L-trace/UAS-drice RNAi; L-CasExpress/+*
Fig 3g	*enGal4 UAS-RFP L-trace/UAS-miRGH; L-CasExpress/+*
Fig 3h	*enGal4 UAS-RFP/L-trace; UAS-puc/L-CasExpress*
Fig 3i	*enGal4 UAS-RFP L-trace/UAS-bsk RNAi; L-CasExpress/+*
Fig 3k	*enGal4/TRE:DsRed*
Fig 3l	*enGal4/TRE:DsRed; UAS-p35/+*
Fig 4b	*enGal4 UAS-RFP/L-trace; UAS-w RNAi/L-CasExpress tubGal80^{ts}*
Fig 4c & h	*enGal4 UAS-RFP/L-trace; UAS-akt1 RNAi/L-CasExpress tubGal80^{ts}*
Fig 4e	*enGal4 UAS-RFP FRTSTOPFRTGFP/+; UAS-w RNAi/tubGal80^{ts}*
Fig 4f	*enGal4 UAS-RFP FRTSTOPFRTGFP/+; UAS-akt1 RNAi/tubGal80^{ts}*
Fig 5a & k	*enGal4 UAS-RFP/L-trace; UAS-dCIZ1 RNAi (BL#28329)/L-CasExpress*
Fig 5b	*enGal4 UAS-RFP/L-trace; UAS-dCIZ1 RNAi (BL#27562)/L-CasExpress*
Fig 5d	*enGal4 UAS-RFP/+; UAS-dCIZ1 RNAi (BL#28329)/+*
Fig 5e	*enGal4 UAS-RFP/+; UAS-dCIZ1 RNAi (BL#27562)/+*
Fig 5g	*enGal4 UAS-RFP L-trace/UAS-dCIZ1:3Xflag; UAS-dCIZ1 RNAi (BL#28329)/L-CasExpress*
Fig 5h	*enGal4 UAS-RFP L-trace/UAS-dCIZ1:3Xflag; UAS-dCIZ1 RNAi (BL#27562)/L-CasExpress*
Fig 6a	*enGal4 UAS-RFP/+; UAS-pten RNAi/+*
Fig 6b	*enGal4 UAS-RFP/L-trace; UAS-pten RNAi/L-CasExpress*
Fig 6d	*enGal4 UAS-RFP L-trace/UAS-miRGH; UAS-pten RNAi/L-CasExpress*
Fig 6g	*enGal4 UAS-RFP L-trace/UAS-pi3k92e RNAi; UAS-pten RNAi/L-CasExpress*
Fig 6j	*enGal4 UAS-RFP/UAS-pi3k92e*
Fig 6k	*enGal4 UAS-RFP/L-trace; UAS-pi3k92e/L-CasExpress*
Fig 6m	*enGal4 UAS-RFP L-trace/UAS-miRGH; UAS-pi3k92e/L-CasExpress*
Fig 6o	*enGal4 UAS-RFP FRTSTOPFRTGFP/+; UAS-myr:akt1/tubGal80^{ts}*
Fig 6p	*enGal4 UAS-RFP/L-trace; UAS-myr:akt1/L-CasExpress tubGal80^{ts}*
Fig 6r	*enGal4 UAS-RFP L-trace/UAS-miRGH; UAS-myr:akt1/L-CasExpress tubGal80^{ts}*
Fig 7a	*enGal4 UAS-RFP L-trace/UAS-pi3k92e; UAS-w RNAi/L-CasExpress*
Fig 7b	*enGal4 UAS-RFP L-trace/UAS-pi3k92e; UAS-dCIZ1 RNAi (BL#28329)/L-CasExpress*
Fig 7c	*enGal4 UAS-RFP L-trace/UAS-w RNAi; UAS-myr:akt1/L-CasExpress Gal80^{ts}*

Fig 7d	*enGal4 UAS-RFP L-trace/UAS-dCIZ1 RNAi (vdrc35344); UAS-myr:akt1/L-CasExpress Gal80^{ts}*
Fig 7e	*enGal4 UAS-RFP L-trace/UAS-dCIZ1 RNAi (vdrc35344); UAS-w RNAi/L-CasExpress Gal80^{ts}*
Fig 8a	*enGal4 UAS-RFP L-trace/UAS-ras^{V12}; UAS-w RNAi/L-CasExpress Gal80^{ts}*
Fig 8c & f	*enGal4 UAS-RFP FRTSTOPFRTGFP/UAS-ras^{V12}; tubGal80ts/+*
Fig 8d	*enGal4 UAS-RFP L-trace/UAS-miRGH; UAS-ras^{V12}/L-CasExpress tubGal80^{ts}*
Fig 8g	*enGal4 UAS-RFP L-trace/UAS-ras^{V12}; UAS-akt1 RNAi/L-CasExpress tubGal80^{ts}*
Fig 8i	*enGal4 UAS-RFP L-trace/UAS-ras^{V12}; UAS-dCIZ1 RNAi (BL#28329)/L-CasExpress tubGal80^{ts}*
Fig 8k & n	*enGal4 UAS-RFP FRTSTOPFRTGFP/UAS-ras^{V12}; tubGal80^{ts}/UAS-dCIZ1 RNAi (BL#28329)*
Fig S1a-c, j-p, r, s	*CasExpress/G-trace; tubGal80^{ts}/+*
Fig S1d-f	*DQVA-Gal4/G-trace; tubGal80^{ts}/+*
Fig S1g-i	*CasExpress/G-trace; dronc^{I29}/dronc^{I29}*
Fig S2a	*enGal4UAS-RFP/L-trace; UAS-p35/L-CasExpress*
Fig S2c	*dcp-1^{prev}/+; L-CasExpress/L-trace*
Fig S2d	*dcp-1^{prev}/dcp-1^{prev}; L-CasExpress/L-trace*
Fig S2f	*CasExpress/G-trace*
Fig S2g	*CasExpress/G-trace; H99/MM2*
Fig S3a	*enGal4 UAS-RFP/L-trace; UAS-n RNAi/L-CasExpress*
Fig S3c	*enGal4 UAS-RFP/+; UAS-n RNAi/+*
Fig S3d	*enGal4 UAS-RFP/L-trace; UAS-ex RNAi/L-CasExpress*
Fig S3f	*enGal4 UAS-RFP/+; UAS-ex RNAi/+*
Fig S3g&i	*enGal4 UAS-RFP/L-trace; UAS-bip2 RNAi/L-CasExpress*
Fig S4a	*enGal4 UAS-RFP/L-trace; UAS-chico RNAi (BL#36665)/L-CasExpress*
Fig S5b	*UAS-DsRed/+; rn-Gal4 UAS-rpr Gal80^{ts}/+*
Fig S5c	*UAS-dCIZ1:3Xflag/+; rn-Gal4 UAS-rpr Gal80^{ts}/+*
Fig S6b	*enGal4 UAS-RFP/L-trace; UAS-inr RNAi/L-CasExpress*
Fig S6c	*enGal4 UAS-RFP/L-trace; UAS-chico RNAi/L-CasExpress*
Fig S6d	*enGal4 UAS-RFP/L-trace; UAS-pi3k92e RNAi/L-CasExpress*
Fig S6e	*enGal4 UAS-RFP/L-trace; UAS-pi3k21b RNAi/L-CasExpress*
Fig S6f	*enGal4 UAS-RFP/L-trace; UAS-pten RNAi/L-CasExpress*
Fig S7a	*UAS-dCIZ1 RNAi (vdrc35344)/+; hh-Gal4/+*
Fig S7b	*UAS-dCIZ1 RNAi (vdrc35344)/UAS-dCIZ1:3Xflag; hh-Gal4/+*

Supplementary table S3. Antibodies

Antibody	Company and Catalog #	Dilution
Rabbit anti-cleaved Dcp-1	Cell Signaling Technologies<br>Cat# 9578S	1:200
Rabbit anti-phosph-Akt1 (Ser473)	Cell Signaling Technologies<br>Cat# 4060S	1:200
Mouse anti-Engrailed	Developmental Studies Hybridoma Bank<br>Cat#4D9	1:10
Mouse anti-Wingless	Developmental Studies Hybridoma Bank<br>Cat#4D4	1:400
goat-anti-rabbit IgG (H+L) Alexa Fluor 488	ThermoFisher Scientific<br>Cat# A-11008	1:200
goat-anti-rabbit IgG (H+L) Alexa Fluor 647	ThermoFisher Scientific<br>Cat# A-21245	1:200
goat-anti-mouse IgG (H+L) Alexa Fluor 488	ThermoFisher Scientific<br>Cat# A-11029	1:200
goat-anti-mouse IgG (H+L) Alexa Fluor 647	ThermoFisher Scientific<br>Cat# A-21235	1:200
goat-anti-rat IgG (H+L) Alexa Fluor 647	ThermoFisher Scientific<br>Cat# A-21247	1:200
mouse anti-FLAG M2	Sigma Aldrich<br>Cat#F1804	1:2000
rabbit anti-Drosophila ICE	Cell Signaling Technologies<br>Cat#13085	1:1000
rabbit anti-Akt	Cell Signaling Technologies<br>Cat#4691T	1:1000
mouse anti-β-actin	Sangon Biotech<br>Cat#D191047	1:1000

Supplementary table S4. Primers used in qPCR.

gene	forward	reverse	source
rp49	GCTAAGCTGTCGCACAAATG	GTTCGATCCGTAACCGATGT	[7]
chico	ACATCAATCGCCGTTTGGACA	GAGAACGATGCCGAATCCAC	DRSC Flyprimer bank
CG8108	GCCACTACGACAACAGGAGT C	CGCGAGATGAGTCATAGCTG C	DRSC Flyprimer bank

Supplementary reference

1. Sun, G. & Irvine, K. D. Regulation of Hippo signaling by Jun kinase signaling during compensatory cell proliferation and regeneration, and in neoplastic tumors. *Dev Biol* **350,** 139–151 (2011).
2. Martín-Blanco, E. *et al.* puckered encodes a phosphatase that mediates a feedback loop regulating JNK activity during dorsal closure in Drosophila. *Genes Dev* **12,** 557–570 (1998).
3. Bunker, B. D., Nellimoottil, T. T., Boileau, R. M., Classen, A. K. & Bilder, D. The transcriptional response to tumorigenic polarity loss in Drosophila. *elife* **4,** (2015).
4. Peterson, C., Carney, G. E., Taylor, B. J. & White, K. reaper is required for neuroblast apoptosis during Drosophila development. *Development* **129,** 1467–1476 (2002).
5. Santabárbara-Ruiz, P. *et al.* ROS-Induced JNK and p38 Signaling Is Required for Unpaired Cytokine Activation during Drosophila Regeneration. *PLoS Genet* **11,** e1005595 (2015).
6. Smith-Bolton, R. K., Worley, M. I., Kanda, H. & Hariharan, I. K. Regenerative growth in Drosophila imaginal discs is regulated by Wingless and Myc. *Dev Cell* **16,** 797–809 (2009).
7. Wang, B. *et al.* The insulin-regulated CREB coactivator TORC promotes stress resistance in Drosophila. *Cell Metab* **7,** 434–444 (2008).
