# Supplementary material for: Akt1 and dCIZ1 promote cell survival from apoptotic caspase activation during regeneration and oncogenic overgrowth
Source: Nat Commun. 2020 Nov 12;11:5726. doi: 10.1038/s41467-020-19068-2 (PMC7664998; doi:10.1038/s41467-020-19068-2)
Supplement: Supplementary file 3 — Reporting Summary [file 41467_2020_19068_MOESM3_ESM.pdf]

nature research

Corresponding author(s): Gongping Sun and Denise Montell

Last updated by author(s): 8/31/2020

Reporting Summary

Nature Research wishes to improve the reproducibility of the work that we publish. This form provides structure for consistency and transparency in reporting. For further information on Nature Research policies, see Authors & Referees and the Editorial Policy Checklist.

Statistics

For all statistical analyses, confirm that the following items are present in the figure legend, table legend, main text, or Methods section.

n/a	Confirmed	
☐	☒	The exact sample size (*n*) for each experimental group/condition, given as a discrete number and unit of measurement
☐	☒	A statement on whether measurements were taken from distinct samples or whether the same sample was measured repeatedly
☐	☒	The statistical test(s) used AND whether they are one- or two-sided<br>*Only common tests should be described solely by name; describe more complex techniques in the Methods section.*
☒	☐	A description of all covariates tested
☐	☒	A description of any assumptions or corrections, such as tests of normality and adjustment for multiple comparisons
☐	☒	A full description of the statistical parameters including central tendency (e.g. means) or other basic estimates (e.g. regression coefficient) AND variation (e.g. standard deviation) or associated estimates of uncertainty (e.g. confidence intervals)
☐	☒	For null hypothesis testing, the test statistic (e.g. *F*, *t*, *r*) with confidence intervals, effect sizes, degrees of freedom and *P* value noted<br>*Give P values as exact values whenever suitable.*
☒	☐	For Bayesian analysis, information on the choice of priors and Markov chain Monte Carlo settings
☒	☐	For hierarchical and complex designs, identification of the appropriate level for tests and full reporting of outcomes
☒	☐	Estimates of effect sizes (e.g. Cohen's *d*, Pearson's *r*), indicating how they were calculated

Our web collection on statistics for biologists contains articles on many of the points above.

Software and code

Policy information about availability of computer code

Data collection	ZEN black (version 14.0, Zeiss) was used for confocal imaging. Bio-rad CFX Manager (version 3.1 Bio-rad) was used to collecting qPCR data.
Data analysis	ImageJ (version 2.0) was used for image analysis and quantification. Prism 6 was used for statistical analysis.

For manuscripts utilizing custom algorithms or software that are central to the research but not yet described in published literature, software must be made available to editors/reviewers. We strongly encourage code deposition in a community repository (e.g. GitHub). See the Nature Research guidelines for submitting code & software for further information.

Data

Policy information about availability of data

All manuscripts must include a data availability statement. This statement should provide the following information, where applicable:

- Accession codes, unique identifiers, or web links for publicly available datasets
- A list of figures that have associated raw data
- A description of any restrictions on data availability

All the raw data supporting the findings of this study are available from the corresponding authors upon request. The source data underlying Figs 1m, 3j, 4d, 4g, 4i, 5c, 5f, 5h, 5i, 5j, 5l, 5m, 6c, 6e, 6f, 6h, 6i, 6l, 6n, 6q, 6s, 7f, 7g, 7h, 8b, 8e, 8h, 8j, 8l, 8m and Supplementary Figs S2b, S2e, S2h, S3b, S3e, S3h, S4b, S5d, S5e, S6g are provided as a Source Data file.
